# Geniposide Combined With Notoginsenoside R1 Attenuates Inflammation and Apoptosis in Atherosclerosis *via* the AMPK/mTOR/Nrf2 Signaling Pathway

**DOI:** 10.3389/fphar.2021.687394

**Published:** 2021-07-07

**Authors:** Xiaoyu Liu, Yuling Xu, Saibo Cheng, Xinghong Zhou, Fenghua Zhou, Peikun He, Fang Hu, Lifang Zhang, Yuyao Chen, Yuhua Jia

**Affiliations:** ^1^ School of Traditional Chinese Medicine, Southern Medical University, Guangzhou, China; ^2^ College of Health, Fujian Medical University, Fuzhou, China

**Keywords:** geniposide, notoginsenoside R1, apoptosis, inflammation, atherosclerosis, AMPK/mTOR/Nrf2 signal pathway

## Abstract

Inflammation and apoptosis of vascular endothelial cells play a key role in the occurrence and development of atherosclerosis (AS), and the AMPK/mTOR/Nrf2 signaling pathway plays an important role in alleviating the symptoms of AS. Geniposide combined with notoginsenoside R1 (GN combination) is a patented supplement for the prevention and treatment of AS. It has been proven to improve blood lipid levels and inhibit the formation of AS plaques; however, it is still unclear whether GN combination can inhibit inflammation and apoptosis in AS by regulating the AMPK/mTOR/Nrf2 signaling pathway and its downstream signals. Our results confirmed that the GN combination could improve blood lipid levels and plaque formation in ApoE^**−/−**^ mice fed with a high-fat diet (HFD), inhibit the secretion of serum inflammatory factors and oxidative stress factors. It also decreased the expression of pyrin domain containing protein 3 (NLRP3) inflammasome-related protein and Bax/Bcl2/caspase-3 pathway-related proteins. At the same time, the GN combination could also inhibit the H_2_O_2_-induced inflammatory response and apoptosis of human umbilical vein endothelial cells (HUVECs), which is mainly related to the activation of the AMPK/mTOR pathway by GN combination, which in turn induces the activation of Nrf2/HO-1 signal. In addition, the above phenomenon could be significantly reversed by dorsomorphin. Therefore, our experiments proved for the first time that the GN combination can effectively inhibit AS inflammation and apoptosis by activating the AMPK/mTOR/Nrf2 signaling pathway to inhibit the NLRP3 inflammasome and Bax/Bcl2/caspase-3 pathway.

## Introduction

The prevalence and mortality of cardiovascular diseases in China is on the rise given the increasing aging population and overall lifestyle changes. Cardiovascular mortality accounts for >40% of the overall mortality, which is higher than that of cancers and other diseases (Liu et al., 2019; Zhao et al., 2019). Therefore, cardiovascular disease, specifically atherosclerotic cardiovascular disease, is still a major cause of mortality, both in China and worldwide (Colantonio and Muntner, 2019; Poller et al., 2020). Atherosclerosis (AS) is the underlying pathological cause of many cardiovascular and cerebrovascular diseases. Increasing evidences confirm that endothelial cell dysfunction-induced inflammation and apoptosis play vital roles in the occurrence and development of AS (Back et al., 2019; Paone et al., 2019). Vascular endothelial cells (VECs) are the single cell layers of arteries, veins and capillaries, which form a barrier between the circulating blood and the wall of blood vessels, and AS begins with dysfunction of VECs (Back et al., 2019; Paone et al., 2019). However, oxidative stress is the key mechanism of vascular endothelial cell injury (Marchio et al., 2019; Yan et al., 2019). When vascular endothelial cells are stimulated by H_2_O_2_ or ox-LDL, several inflammatory factors and adhesion molecules, such as interleukin-6 (IL-6), tumor necrosis factor-α (TNF-α), vascular cell adhesion molecule 1 (VCAM-1) and intercellular cell adhesion molecule-1 (ICAM-1), are secreted that can promote the aggregation, migration, and deposition of monocytes into the vascular endothelium (Galkina and Ley, 2009). Subsequently, monocytes are transformed into macrophages by lipid phagocytosis and eventually transformed into foam cells. These foam cells gradually accumulate to form lipid plaques, and the necrosis and apoptosis of foam cells lead to necrosis of the lipid core, thereby activating a vascular inflammatory response, resulting in apoptosis, abnormal lipid metabolism, and autophagy. In addition, H_2_O_2_ can also reduce the activity of superoxide dismutase (SOD) in vascular endothelial cells, resulting in the inability to clear reactive oxygen species (ROS) in time, and ROS can activate the apoptotic response of caspase in cells, leading to cell apoptosis (Shaw et al., 2021). Therefore, inhibiting the inflammatory response and apoptosis of vascular endothelial cells induced by H_2_O_2_ is of great significance for the prevention and treatment of AS.

Nuclear factor erythroid 2-related factor 2 (Nrf2) belongs to the transcriptional activator family, which is a central transcription factor of the endogenous antioxidant pathway (Back et al., 2019; Paone et al., 2019). Combined with antioxidant response elements, Nrf2 regulated the protein expression level of a series of antioxidant factors, including heme oxygenase-1 (HO-1), SOD, glutathione (GSH), catalase (CAT), and quinone oxidoreductase-1 (NQO-1), thus playing a significant role in anti-oxidant stress (Loboda et al., 2016). Current evidence suggests that the Nrf2/HO-1 pathway plays an anti-AS role by inhibiting inflammatory response and apoptosis (Chen et al., 2019). Adenosine 5′- monophosphate activated protein kinase (AMPK) is a serine/threonine protein kinase, which is a key energy sensor of cell metabolism (Day et al., 2017). Studies have pointed out that the stability of AS plaques is closely related to the activation of AMPK (Liang et al., 2018). AMPK can inhibit the phosphorylation of mammalian target of rapamycin (mTOR), and thus participate in the pathological process of inflammation, apoptosis, oxidative stress, and autophagy (Kazyken et al., 2019; Kimura et al., 2020; Sun et al., 2020). Therefore, activation of the AMPK/mTOR signaling pathway is associated with anti-inflammatory and anti-apoptotic effects. Meanwhile, there is a number of evidence that AMPK promotes Nrf2 nuclear accumulation by directly phosphorylating its ser550 residue, and the mTOR can trigger senescence in HUVECs by inhibiting Nrf2 transcription (Joo et al., 2016; Yang et al., 2018). Hence, the activation of the AMPK/mTOR pathway may contribute to activation of the Nrf2 pathway, which can inhibit the inflammatory response and apoptosis of AS.

Geniposide (GP) is an iridoid glycoside extracted from the dried fruits of Gardenia jasminoides Ellis, which is its main active substance. Recent studies have found that GP could inhibit the occurrence and development of AS by inhibiting inflammation, alleviating lipid metabolism disorder, regulating macrophage polarization and enhancing autophagy (Cheng et al., 2019; Jin et al., 2020; Liu et al., 2020; Xu et al., 2020). Notoginsenoside R1 (NR) is a characteristic component of traditional Chinese medicine Panax notoginseng, which have been clinically used to treat cardiovascular and cerebral vascular diseases in China due to its good effect in controlling internal and external bleeding, improving blood stasis and reducing blood pressure (Wang et al., 2016; Yang et al., 2020). Numerous studies have reported that notoginsenoside R1 can inhibit inflammation, apoptosis and oxidative stress and promote angiogenesis (Jia et al., 2014; Zhao et al., 2020; Zhong et al., 2020). Therefore, GP and NR can alleviate the occurrence and development of AS. GN combination is a patented drug combination that has been approved for the prevention and treatment of AS (Patent No.: CN201810425740.8). It has been reported that GP and NR are the main components of Tongluo Jiunao injection, which are mainly used in the treatment of ischemic stroke and vascular dementia in clinic (He et al., 2013). However, the specific mechanism by which this GN combination improves AS is still not clear. Therefore, we used molecular biology experiments to explore the protective effect of GN combination on ApoE^**−/−**^ mice fed with high-fat diet (HFD) and H_2_O_2_-induced HUVECs and the regulatory mechanism of the AMPK/mTOR/Nrf2 signaling pathway on inflammatory response and apoptosis.

## Materials and Methods

### Ethical Review

The animal experiments in this study were approved by the Southern Medical University Experimental Animal Ethics Committee (No.: L2018095).

### Materials

GP (24512-63-8) and NR (80418-24-2) were purchased from Shanghai Yuanye Biotechnology Co., Ltd. (Shanghai, China). Acadesine (AICAR, HY-13417) and dorsomorphin (HY-13418A) were purchased from MedChemExpress (New Jersey, NJ, United States). intracellular pH fluorescence probe (BCECF-AM, S1006) and 2′,7′-dichlorodihydrofluorescein diacetate (DCFH-DA) probe (S0033S) were purchased from Beyotime Biotechnology Co., Ltd. (Shanghai, China). The serum total cholesterol (TC, A111-1-1), triglyceride (TG, A110-1-1), high-density lipoprotein cholesterol (HDL-C, A112-1-1), low-density lipoprotein cholesterol (LDL-C, A113-1-1), malondialdehyde (MDA, A003-1-2), SOD (A001-3-2) and glutathione (GSH, A006-2-1) kits were purchased from Nanjing Jiancheng Bioengineering Institute (Nanjing, China). IL-6 (BS-E8682M1, BS-E3973H1), TNF-α (BS-E9347M1, BS-E5364H1), IL-8 (BS-E8685M1) and IL-15 (BS-E8662M1) ELISA kits were purchased from Jiangsu Boshen Biotechnology Co., Ltd. (Nanjing, China). The PrimeScriptTM RT reagent kit with gDNA Eraser (RR047A) and TB GreenTM Premix Ex TaqTM Ⅱ (RR820A) were purchased from TaKaRa Biotechnology Co., Ltd. (Dalian, China). The primary antibodies for AMPK (10929-2-AP), Caspase-1 (22915-1-AP), Nrf2 (16396-1-AP), Interleukin-1β (IL-1β, 16806-1-AP), Interleukin-18 (IL-18, 10663-1-AP) and NLRP3 (19771-1-AP) were purchased from Proteintech (Pennsylvania, PA, United States), and those for p-AMPK (2537S), mTOR (2983S), p-mTOR (5536S), VCAM-1 (39036S), ICAM-1 (67836S), Bax (14796S), and Bcl-2 (3498S) were purchased from Cell Signaling Technology (Danvers, United States). The primary antibodies for NOX2 (bs-3889R) and p22phox (bs-3879R) were purchased from Bioss Biotechnology Co., Ltd. (Beijing, China). HRP-labeled goat anti-rabbit IgG (G1213) was purchased from Wuhan Seville Biotechnology Co., Ltd. (Wuhan, China); the BCA protein concentration determination kit (23325) was purchased from Thermo Fisher Scientific (Bedford, MA, United States); the ECL luminescent solution (32109) and PVDF membrane (R8MA0424) were purchased from Millipore Corporation (Bedford, MA, United States). All the primers were synthesized by Sangon Biotech Co., Ltd. (Shanghai, China).

### *In Vivo* Experiments in Mice

Eight-week-old male ApoE^**−/−**^ mice (*n* = 10) were purchased from Changzhou Cavens Experimental Animal Co., Ltd. (Jiangsu, China), and were randomly divided into a model control group (Mo, *n* = 10), geniposide group (GP, *n* = 10), notoginsenoside R1 group (NR, *n* = 10), and GP + NR group (GN, *n* = 10). Another 10 male C57BL/6J mice of the same age were selected as the control group (Con). All mice were housed in a specific-pathogen-free animal room under constant temperature and humidity conditions. ApoE^**−/−**^ mice were fed an HFD for 12 weeks to establish the AS model, while the Con group mice were fed a normal diet. After 12 weeks, the GP, NR, and GN groups were respectively given geniposide (50 mg kg^−1^), Panax notoginseng R1 (50 mg kg^−1^) and geniposide + notoginsenoside R1 (50 + 50 mg kg^−1^) by intraperitoneal injection for 12 weeks (Xiao et al., 2018; Xu et al., 2020), and the remaining control mice were treated with the same volume of normal saline. After the last administration, the body weight and fasting blood glucose of all mice were detected. All mice were euthanized by intraperitoneal injection of 0.1% Pentobarbital Sodium and aortic tissue and serum samples were collected for subsequent experiments.

### Cell Culture, Grouping, and Drug Administration

Human umbilical vein endothelial cells (HUVECs) were obtained from Shanghai Zhong Qiao Xin Zhou Biotechnology Co., Ltd. (Shanghai, China), which was cultured in high-glucose Dulbecco's Modified Eagle Medium (DMEM) containing 10% fetal bovine serum (FBS) and 1% penicillin-streptomycin at 37°C in an incubator containing 5% CO_2_. Human acute monocytic leukemia cell line (THP-1) was preserved in molecular biology laboratory, School of Traditional Chinese Medicine, Southern Medical University, which was cultured in RPMI 1640 medium with 10% FBS and 1% penicillin-streptomycin. All other conditions were the same as for HUVECs. The experiment cells were from the 4 to 10 passages.

### MTT Assay

Briefly, HUVECs in logarithmic phase were collected and inoculated into 96-well plates with 10^5^ cells/well. After adding concentration-gradient H_2_O_2_, GP, or NR to each well, HUVECs were incubated in 5% CO_2_ and 37°C for 24 h. Then, 20 μl MTT was added to each well for 4 h and 150 μl DMSO was added. Finally, the absorbance value (OD) of each well was measured at 490 nm by using a microplate reader.

### Serum Lipids

After anesthetic induction, blood samples were collected and stored at 4°C for 2–3 h, and then centrifuged at 1,000 × g for 15 min to collect the supernatant. Finally, serum TC, TG, HDL-C, and LDL-C levels in mice were determined by ELISA according to the manufacturer’s instructions.

### Hematoxylin-Eosin, Masson Staining and Oil Red O Staining

Aortic sinus tissue was immersed in formalin overnight at room temperature. The next day, the samples were dehydrated with gradient alcohol and treated with xylene. Then, the samples were embedded in conventional paraffin and sectioned intermittently to a thickness of 4 μm. HE staining, Masson staining and Oil red O staining were performed to observe the pathological changes.

### Enzyme-Linked Immunosorbent Assay

MDA, SOD, GSH, IL-6, TNF-α, IL-8 and IL-15 in mice and cell supernatant were detected by ELISA. Briefly, all the samples and reagents were stored at room temperature for 20 min, and then the standard and sample were added to the strip, followed by 100 μl of horseradish peroxidase-labeled antibody and incubated for 60 min at 37°C. The plates were washed five times. Then, 50 μl of substrate A and B were added to each well and incubated for 15 min at 37°C in the dark. Finally, 50 μl of termination solution was added to each well, and the OD value of each well was determined at 450 nm.

### Monocyte Adhesion Test

HUVECs in logarithmic growth phase were inoculated into 6-well plates at the rate of 1 × 10^6^ cells/well and treated with GP, NR, and GN for 24 h. THP-1 cells were stained with 5 μmol/L BCECF-AM for 0.5 h, washed thrice with PBS, and then resuspended in DMEM without FBS. THP-1 was seeded into HUVECs at a density of 2 × 10^5^ cells/L and incubated in a 5% CO_2_ incubator at 37°C for 2 h. The 6-well plate was taken out and washed thrice with PBS. The non-adherent cells were washed out, and the plate was observed under an inverted microscope. Three different fields of vision were observed for each group, and the fluorescent spots in these images were counted using ImageJ software (National Institutes of Health, Bethesda, MD, United States). The above experiment was repeated three times.

### Flow Cytometry

The cells in logarithmic growth phase were inoculated into 6-well plates at a concentration of 4 × 10^4^ cells/well. After treatment with different concentrations of drugs for 24 h, the cells were digested with trypsin without EDTA and centrifuged at 500 g for 5 min. After two washes with pre-cooled PBS, the cells were resuspended with 1 × 10^6^ cells·ml^−1^ in 1 × binding buffer. Then, 5 μl annexin V-FITC and 5 μl 7-AAD were added for 15 min and incubated in the dark. Finally, 400 μl × binding buffer was added into each tube of cells, and the apoptosis rate was measured by flow cytometry.

### Determination of Intracellular Reactive Oxygen Species

The level of ROS in HUVECs was detected by DCFH-DA probe. The cells in logarithmic growth phase were inoculated into 6-well plates at a concentration of 4 × 10^4^ cells/well. After treatment with different concentrations of drugs for 24 h, the cells were incubated with 10 μM DCFH-DA for 60 min at 37°C. The fluorescence microscopy was used to observe the level of green fluorescence in HUVECs.

### Western Blot

The protein expression levels of AMPK, p-AMPK, mTOR, p-mTOR, Nrf2, caspase-1, IL-1β, IL-18, NLRP3, VCAM-1, ICAM-1, caspase-3, NOX2, p22phox, Bax, and Bcl-2 in aortic tissue and HUVECs were detected by WB. The total protein was extracted by RIPA lysate with phenylmethanesulfonyl and phosphatase inhibitor, and the bicinchoninic acid (BCA) assay was used for protein quantification. After electrophoresis and membrane transfer, 5% bovine serum albumin (BSA) was used to block the protein for 2 h at room temperature. The primary antibody was incubated at 4°C overnight, and the secondary antibody was incubated at 37°C for 1 h. The expression level of protein was analyzed using ImageJ software.

### RT-qPCR

RT-qPCR was used to detect the mRNA expression levels of *AMPK*, *mTOR*, *Nrf2*, and GAPDH in HUVECs. Briefly, total RNA was extracted from tissues and cells, cDNA was obtained by reverse transcription according to the instructions of PrimeScript™ RT Reagent Kit with gDNA eraser. The designed primers and GAPDH primers were used to amplify the cDNA. The reaction cocktail comprised 0.4 μl Rox Reference Dye, 0.8 μl upstream primer, 0.8 μl downstream primer, 10 μl SYBR premix ex Taq II, 6 μl enzyme free water, and 2 μl cDNA. The amplification conditions were as follows: pre-denaturation at 95°C for 30 s and 40 cycles of denaturation at 95°C for 15 s and annealing/extension at 60°C for 20 s. Finally, the melting curve was calculated. The expression of targets were normalized by *GAPDH* expression, and 2^(−ΔΔCT)^ method was used to calculated the fold changes of mRNA expression. Each sample was made with 2 holes. The primer sequences are shown in Table 1.

**TABLE 1:** The primer sequences used in qRT-PCR analysis.

Gene	Primer sequence (5′-3′)
*AMPK*	Forward: ACC​AGG​TCA​TCA​GTA​CAC​CAT​C
	Reverse: GTT​GGA​ACA​GAC​GCC​GAC​TT
*mTOR*	Forward: TCG​CTG​AAG​TCA​CAC​AGA​CC
	Reverse: CTT​TGG​CAT​ATG​CTC​GGC​AC
*Nrf2*	Forward: TCA​GAA​ACC​AGT​GGA​TCT​GCC
	Reverse: GGA​ATG​TCT​GCG​CCA​AAA​GC
*GAPDH*	Forward: ACA​ACT​TTG​GTA​TCG​TGG​AAG​G
	Reverse: GCC​ATC​ACG​CCA​CAG​TTT​C

### Immunofluorescence

The cells in the logarithmic growth phase were inoculated into 6-well plates at a concentration of 4 × 10^4^ cells/well. After 24 h of treatment, the following steps were carried out: First, the cells were washed thrice with PBS (5 min/wash), followed by blocking with 5% normal goat serum for 30 min; the excess liquid was discarded. Second, the corresponding first antibody was added and incubated overnight at 4°C, and 0.01 mol/L PBS was used as the negative control. Third, after cleaning with PBS, fluorescent-labeled secondary antibody was added and incubated for 1 h at 37°C. Fourth, after wash with PBS three times, 5 min each time, DAPI staining was performed for 10 min. Finally, after another washing with PBS, the film was sealed with an anti-fluorescence quenching agent and observed under the Upright fluorescence microscope (DM3000, Frankfurt, Germany).

### Statistics

The results were expressed as mean ± standard deviation. If the data of multiple groups were in accordance with normal distribution and homogeneity of variance, one-way ANOVA was selected; if not, Kruskal–Wallis H test and Wilcoxon rank sum test were used. *p* < 0.05 was considered to indicate statistically significant differences.

## Results

### GN Combination Alleviated Disorders of Lipid Metabolism and Aortic Plaque in HFD-Fed ApoE^−/−^ Mice

**FIGURE 1:**
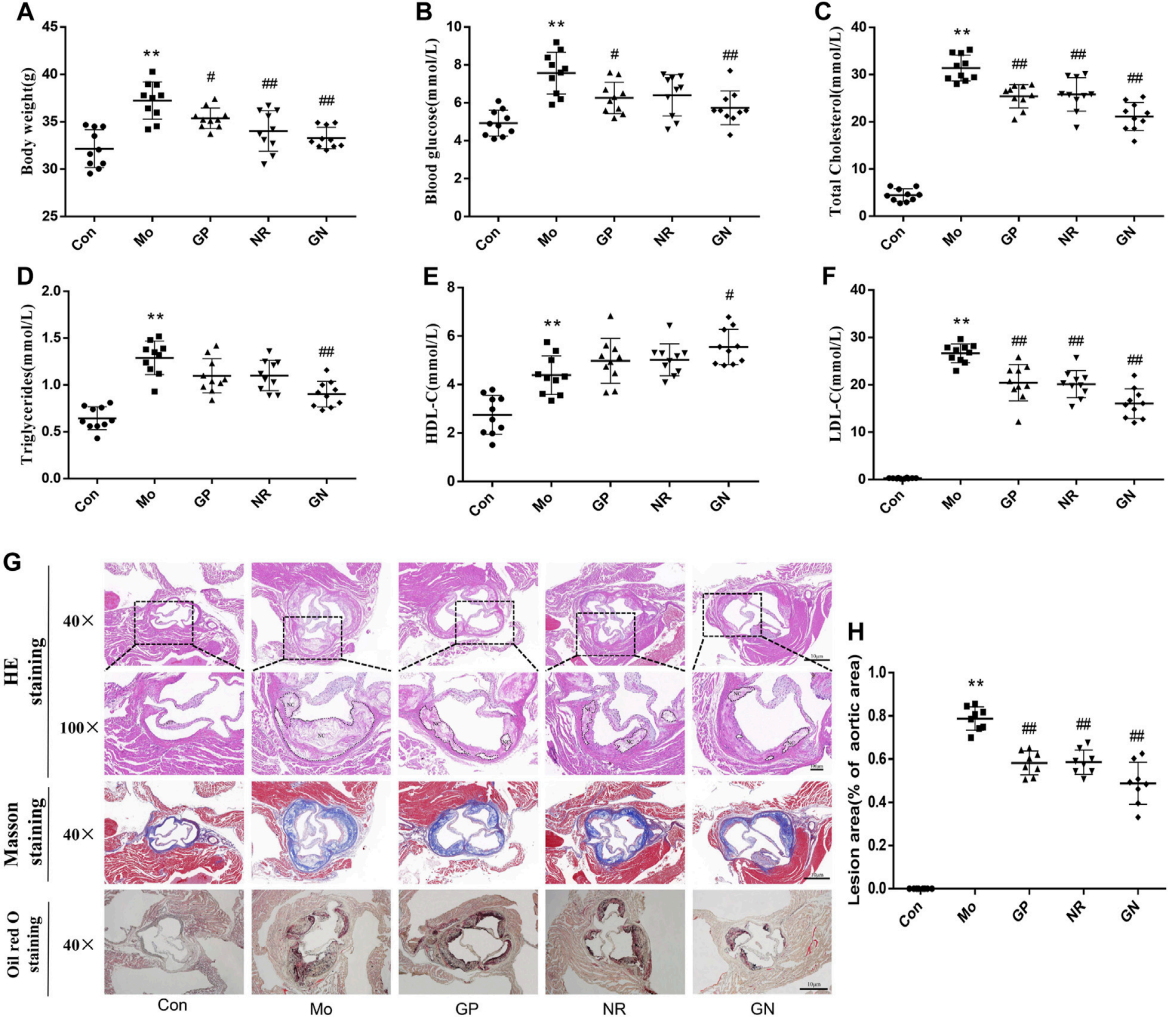
GN combination alleviated disorders of lipid metabolism and aortic root plaque in HFD-fed ApoE^−/−^ mice. ApoE^−/−^ mice were randomly divided into a model control group (Mo), geniposide group (GP), notoginsenoside R1 group (NR), and GP + NR group (GN), another 10 male C57BL/6J mice were selected as the control group (Con). **(A)** Effect of GN combination on body weight in ApoE^−/−^ mice. **(B)** Effect of GN combination on blood glucose in ApoE^−/−^ mice (*n* = 10). **(C–F)** Effect of GN combination on serum TC, TG, HDL-C, and LDL-C levels (*n* = 10). **(G)** HE staining, Masson staining and Oil red O staining of aortic sinus tissue sections in ApoE^**−/−**^ mice (scale bar, 10 μm, 40×; 100 μm, 100 ×; NC, Necrotic core). **(H)** The atherosclerotic lesion areas were quantified by ImageJ software (*n* = 8). All the data are presented as the mean ± SD. *p < 0.05 vs. control group, **p < 0.01 vs. control group, ^#^ *p* < 0.05 vs. model group, ^##^ *p* < 0.01 vs. model group.

To explore the effect of GN combination on AS, we detected the physiological indices of HFD-fed ApoE^**−/−**^ mice. Compared with the control group, the model group showed significantly increased body weight and blood glucose, TC, TG, HDL-C, and LDL-C levels. However, GP, NR, and GN could reduce the body weight and blood glucose, TC, TG, and LDL-C levels of ApoE^**−/−**^ mice and increase the HDL-C levels, and the effect of GN group is more obvious (Figures 1A–F). The pathological morphology of aortic tissue was examined by optical microscopy. The staining results of pathological section showed that compared with the control group, a large amount of plaque appeared in the aortic root in the model group. It indicated that feeding ApoE^**−/−**^ mice with HFD could successfully replicate atherosclerosis model. After treatment with GP, NR or GN, the lesion area of the aortic root plaque were lower than that in the model group (Figures 1G,H). Large area of necrotic core can increase plaque vulnerability, even lead to plaque rupture and acute coronary syndrome. The result of HE and Masson staining showed that the area of necrotic core in aortic plaque in GP, NR or GN treatment group was significantly reduced compared with that in model group, and the area of collagen fiber was increased. Moreover, GN combination had a better effect on AS than GP or NR alone.

### GN Combination Inhibited the Inflammatory Response and Apoptosis in HFD-Fed ApoE^**−/−**^ Mice

**FIGURE 2:**
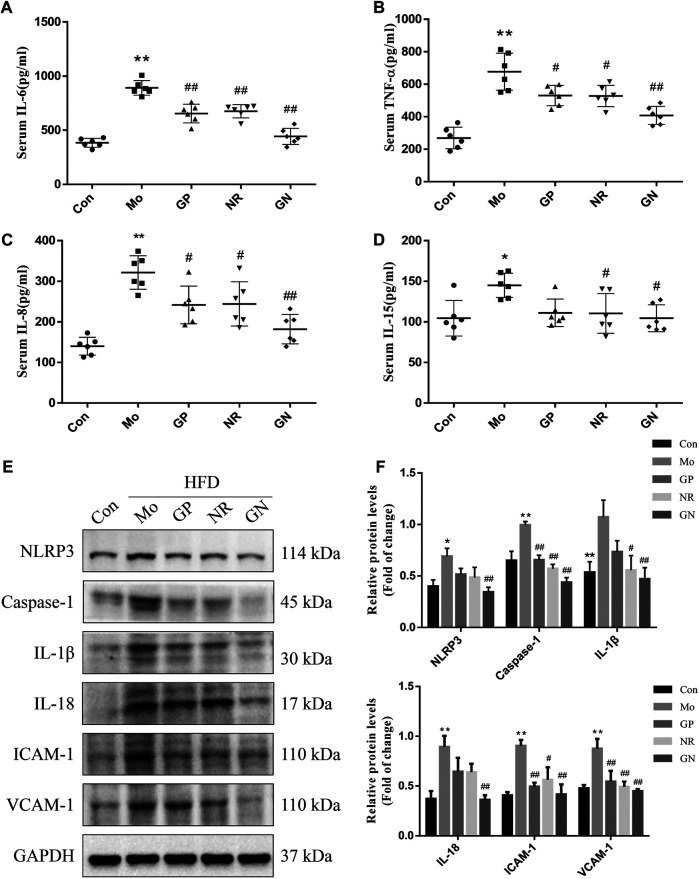
GN combination inhibited inflammatory response in HFD-fed ApoE^−/−^ mice**.** Serum level of IL-6. **(A)**, TNF-α **(B)**, IL-8 **(C)** and IL-15. **(D)** (*n* = 6). **(E)** The expression of NLRP3, caspase-1, IL-1β, IL-18, ICAM-1, VCAM-1, and GAPDH was determined by WB. **(F)** The relative protein levels of NLRP3, caspase-1, IL-1β, IL-18, ICAM-1, and VCAM-1 were normalized to GAPDH (*n* = 3). All data are shown as the mean ± SD. *p < 0.05 vs. control group, **p < 0.01 vs. control group, ^#^ *p* < 0.05 vs. model group, ^##^ *p* < 0.01 vs. model group.

To analyze the effect of GN combination on the inflammatory response of HFD-fed ApoE^−/−^ mice, we used ELISA to detect the concentrations of IL-6, TNF-α, IL-8 and IL-15 in the serum, and WB to detect the expression of NLRP3, caspase-1, IL-1β, IL-18, ICAM-1, and VCAM-1 in aortic tissue. ELISA results showed that HFD could induce increases in serum IL-6, TNF-α, IL-8 and IL-15 of ApoE^**−/−**^ mice, while GP, NR, and GN combination treatment could reduce the concentration of IL-6, TNF-α, IL-8 and IL-15, especially the GN combination (Figures 2A–D). At the same time, HFD could significantly promote the protein expression of NLRP3, caspase-1, IL-1β, IL-18, ICAM-1, and VCAM-1 in the aortic tissue of ApoE^**−/−**^ mice; these effects were significantly alleviated by GN combination (Figures 2E,F).

**FIGURE 3:**
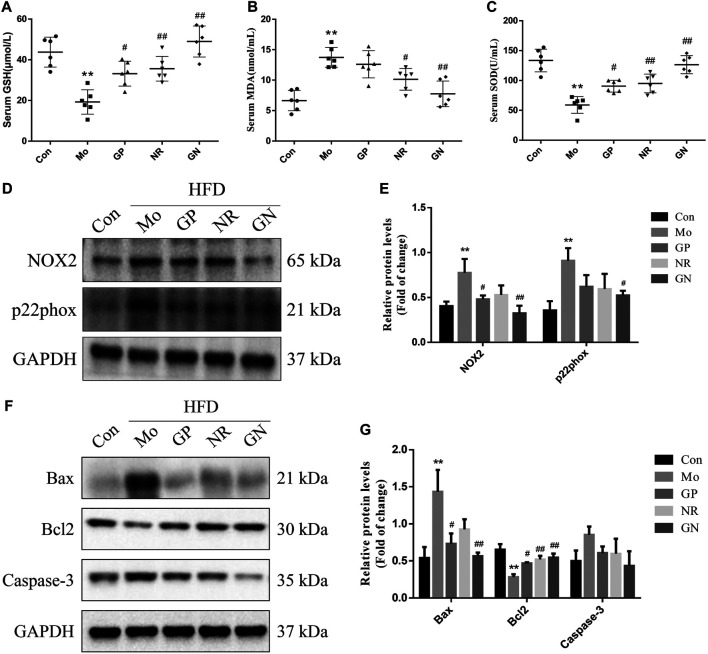
GN combination inhibited apoptosis in HFD-fed ApoE^−/−^ mice. Serum level of GSH. **(A)**, MDA **(B)** and SOD. **(C)** (*n* = 6). **(D)** The expression of NOX2, p22phox and GAPDH was determined by WB. **(E)** The relative protein levels of NOX2 and p22phox were normalized to GAPDH (*n* = 3). **(F)** The expression of caspase-3, Bax, Bcl2, and GAPDH was determined by WB. **(G)** The relative protein levels of caspase-3, Bax, and Bcl2 were normalized to GAPDH (*n* = 3). All data are shown as the mean ± SD. *p < 0.05 vs. control group, **p < 0.01 vs. control group, ^#^ *p* < 0.05 vs. model group, ^##^ *p* < 0.01 vs. model group.

Since endothelial cell apoptosis plays an important role in the development of AS, we explored whether GN combination can inhibit the apoptotic response induced by HFD in ApoE^**−/−**^ mice. Oxidative stress is an important factor leading to endothelial cell apoptosis. Therefore, we first detected the levels of GSH, MDA, and SOD in the serum of ApoE^**−/−**^ mice. Compared with the normal control group, the model group showed a nearly 3-fold and 2-fold decrease in serum levels of GSH and SOD, respectively, while MDA increased about 2-fold (Figures 3A–C). GP, NR, and GN combination significantly increased the activities of GSH and SOD and decreased the level of MDA, especially in the GN combination group. NOX2 is the main source of ROS in blood vessels, while p22pox is an important subunit in maintaining the activity of NOX2 (Violi et al., 2017). Our results showed that the over-expressions of NOX2 and p22phox protein induced by HFD in aortic tissue were evidently attenuated after treated with GN combination (Figures 3D,E). Further, HFD could increase the expression of Bax and caspase-3 in the aorta of mice and reduce the expression of Bcl2; whereas, GN combination showed an obvious opposite effect (Figures 3F,G). Therefore, GN combination inhibited inflammatory response and apoptosis in HFD-fed ApoE^**−/−**^ mice.

### GN Combination Activated the AMPK/mTOR/Nrf2 Signaling Pathway in Aortic Tissue of ApoE^**−/−**^ Mice

**FIGURE 4:**
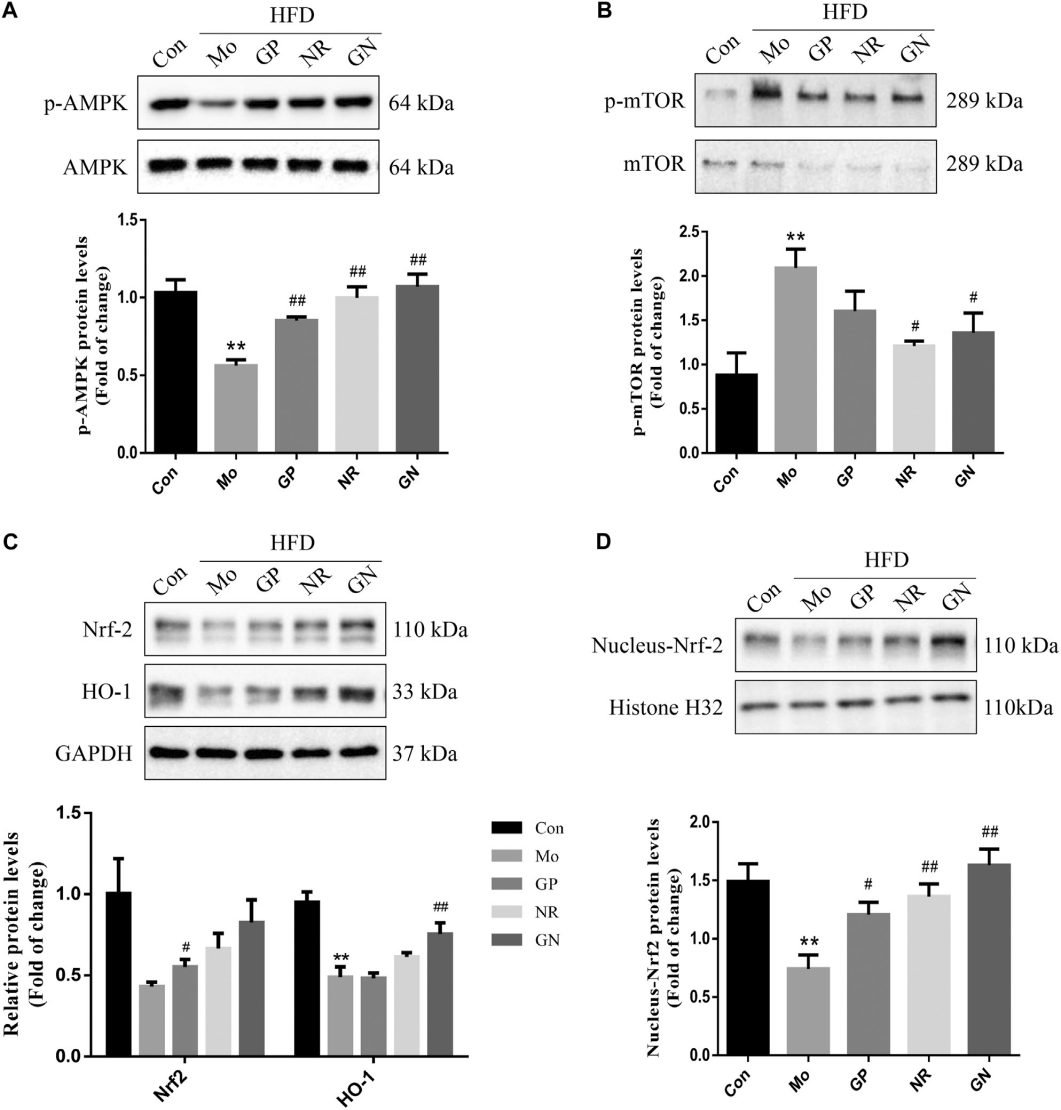
GN combination activated the AMPK/mTOR and Nrf2 signaling pathway in aortic tissue of ApoE^−/−^ mice. The expression of p-AMPK, AMPK. **(A)**, *p*-mTOR, mTOR. **(B)**, t-Nrf-2, HO-1, GAPDH. **(C)**, nuclear-Nrf-2, and histone H32. **(D)** in aortic tissue of ApoE^−/−^ mice was determined by WB, and all the relative protein levels were normalized to GAPDH. All the data are presented as the mean ± SD, *n* = 3. *p < 0.05 vs. control group, **p < 0.01 vs. control group, ^#^ *p* < 0.05 vs. model group, ^##^ *p* < 0.01 vs. model group.

Next, we investigated whether GN combination could activate the AMPK/mTOR/Nrf2 pathway *in vivo*. The WB results showed that HFD could reduce the phosphorylation of AMPK protein and increase the phosphorylation of mTOR protein in the aortas of ApoE^**−/−**^ mice; however, these effects were reversed by GN combination (Figures 4A,B). Nevertheless, the effect of NR used alone was better than that of GN combination in reducing the phosphorylation level of mTOR protein. At the same time, GN combination could effectively induce the expression of Nrf2 and HO-1 in ApoE^**−/−**^ mice. Moreover, the protein expression of nuclear Nrf2 was increased in the GN group **(** Figures 4C,D **)**.

### GN Combination Alleviated H_2_O_2_-Induced Human Umbilical Vein Endothelial Cells Damage *In Vitro*

**FIGURE 5:**
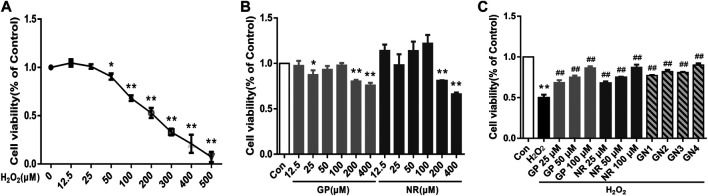
GN combination alleviated *in vitro* H_2_O_2_-induced damage to HUVECs. **(A)** Effects of different concentrations of H_2_O_2_ on the cell viability of HUVECs. **(B)** Effects of different concentrations of GP or NR on the cell viability of HUVECs. **(C)** Effects of different concentrations of GP, NR, or GN combination on the cell viability of HUVECs. GN1: 50 μM GP plus 50 μM NR; GN2: 50 μM GP plus 100 μM NR; GN3: 100 μM GP plus 50 μM NR; GN4: 100 μM GP plus 100 μM NR. All the data are presented as the mean ± SD, *n* = 3. *p < 0.05 vs. control group, **p < 0.01 vs. control group, ^#^ *p* < 0.05 vs. model group, ^##^ *p* < 0.01 vs. model group.

To evaluate the effects of different concentrations of H_2_O_2_, GP, NR, and GN combination on HUVECs, cell viability was determined using the MTT assay. First, HUVECs were treated with H_2_O_2_ at concentrations of 0, 12.5, 25, 50, 100, 200, 300, 400, and 500 μM for 24 h. As shown in Figure 5A, the cell viability decreased with increasing H_2_O_2_ concentration, and the cell survival rate was 52.86% at the concentration of 200 μM H_2_O_2_. Therefore, HUVECs treated with 200 μM H_2_O_2_ for 24 h was used to establish the cell model of inflammation and apoptosis in this study. Second, we selected 12.5, 25, 50, 100, 200, and 400 μM GP or NR to treat HUVECs for 24 h without the intervention of H_2_O_2_. The results showed that the cell viability increased at first and then decreased with increasing drug concentration. The cell viability was the highest at a drug concentration of 100 μM (Figure 5B). Therefore, the optimal concentration of the drug in this study did not exceed 100 μM. Finally, we investigated whether GP, NR, or GN combination could improve the H_2_O_2_-induced damage to HUVECs. The GP, NR, and GN combination groups showed greater cell viability than the H_2_O_2_ group, and 100 μM GP plus 100 μM NR group showed the best effect (Figure 5C). Therefore, 100 μM GP and 100 μM NR were selected as the optimal concentrations for drug intervention.

### GN Combination Ameliorated H_2_O_2_-Induced Inflammatory Response in Human Umbilical Vein Endothelial Cells

**FIGURE 6:**
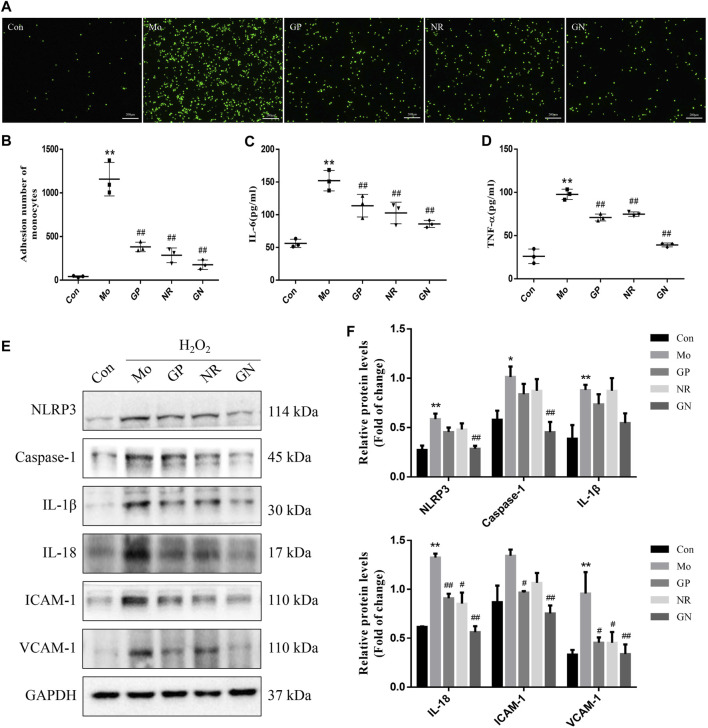
GN combination ameliorated H_2_O_2_-induced inflammatory response in HUVECs. The cells were randomly divided into five groups: Normal control group (Con), Model group (Mo), Geniposide treatment group (GP), Notoginsenoside R1 treatment group (NR) and Geniposide + Notoginsenoside R1 treatment group (GN). **(A)** Monocyte adhesion test: THP-1 cells adhered to HUVECs are seen as green fluorescence (scale bar: 200 μm, 200×). **(B)** The number of THP-1 cells adhered to HUVECs. **(C)** The concentration of IL-6 in cell supernatants. **(D)** The concentration of TNF-α in cell supernatants. **(E)** The expression of NLRP3, caspase-1, IL-1β, IL-18, ICAM-1, VCAM-1, and GAPDH in HUVECs was determined by WB. **(F)** The relative protein levels of NLRP3, caspase-1, IL-1β, IL-18, ICAM-1, and VCAM-1 were normalized to GAPDH. All data are shown as the mean ± SD, *n* = 3. *p < 0.05 vs. control group, **p < 0.01 vs. control group, ^#^ *p* < 0.05 vs. model group, ^##^ *p* < 0.01 vs. model group.

Based on the experimental results *in vivo*, we further explored whether GN combination could inhibit H_2_O_2_-induced inflammatory response in HUVECs. The results of the monocyte adhesion test showed that the number of THP-1 cells adhered to HUVECs induced by H_2_O_2_ for 24 h was significantly higher than that of the normal control cells; GP, NR, and GN combination could reduce the number of adhered THP-1 cells (Figures 6A,B). At the same time, H_2_O_2_ significantly induced HUVECs to secrete IL-6 and TNF-α, while GP, NR, and GN combination inhibited H_2_O_2_-induced IL-6 and TNF-α (Figures 6C,D). We also detected the expression of NLRP3, caspase-1, IL-1β, IL-18, ICAM-1, and VCAM-1 by WB. The results showed that GN combination could significantly inhibit the expression of inflammation-related proteins (Figures 6E,F). These results suggest that GN combination inhibits the inflammatory response of H_2_O_2_-induced HUVECs.

### GN Combination Ameliorated H_2_O_2_-Induced Apoptosis in Human Umbilical Vein Endothelial Cells

**FIGURE 7:**
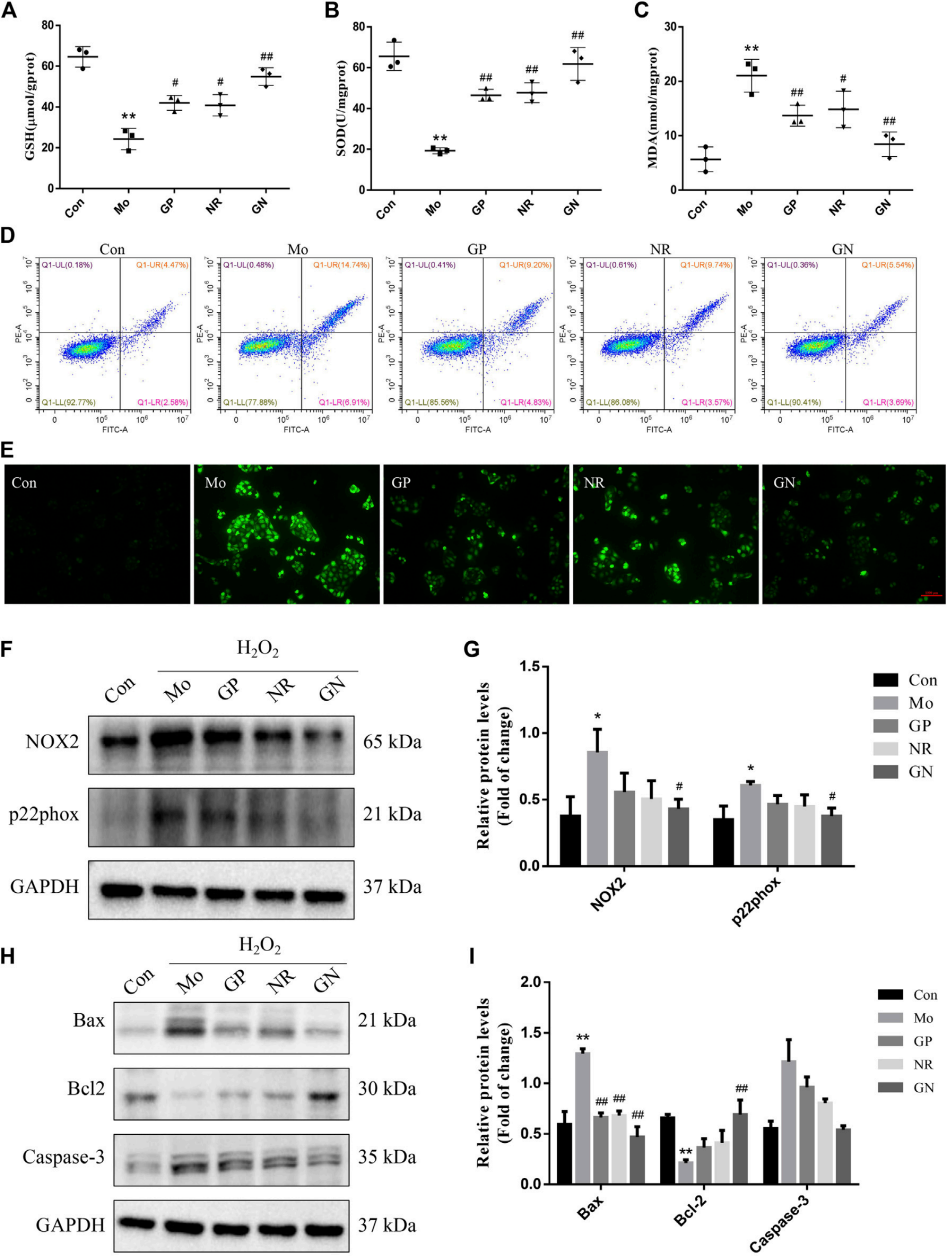
GN combination ameliorated H_2_O_2_-induced apoptosis in HUVECs. **(A)** The concentration of GSH in cell supernatants. **(B)** The concentration of SOD in cell supernatants. **(C)** The concentration of MDA in cell supernatants. **(D)** Representative images of cellular apoptosis detected by flow cytometry. **(E)** ROS generation induced by H_2_O_2_ in HUVECs on fluorescence microscopy (scale bar: 1,000 μm, 100×). **(F)** The expression of NOX2, p22phox and GAPDH in HUVECs was determined by WB. **(G)** The relative protein levels of NOX2 and p22phox were normalized to GAPDH. **(H)** The expression of Bax, Bcl2, caspase-3 and GAPDH in HUVECs was determined by WB. **(I)** The relative protein levels of caspase-3, Bax and Bcl2 were normalized to GAPDH. All the data are presented as the mean ± SD, *n* = 3. *p < 0.05 vs. control group, **p < 0.01 vs. control group, ^#^ *p* < 0.05 vs. model group, ^##^ *p* < 0.01 vs. model group.

Next, we investigated the effect of GN combination on H_2_O_2_-induced apoptosis *in vitro*. After induced by H_2_O_2_, the activities of GSH and SOD in the cell supernatant decreased, while the concentration of MDA increased. GP, NR, and GN combination could reverse the above effects, especially GN combination (Figures 7A–C). The flow cytometry results showed that compared with the controls, H_2_O_2_ treatment significantly increased the apoptosis rate of HUVECs, while treatment with GN combination reduced the H_2_O_2_-induced apoptosis percentage of HUVECs (Figure 7D). As shown in Figure 7E, compared with model group, GN combination markedly decreased the ROS level in HUVECs. In addition, GN combination also inhibited the over-expressions of NOX2 and p22phox induced by H_2_O_2_ in HUVECs (Figures 7F,G). Furthermore, the WB results showed that compared with the model group, the GN group showed decreased expression of Pro apoptotic protein Bax and caspase-3 but increased expression of anti-apoptotic protein Bcl2 (Figures 7H,I). Therefore, it was confirmed that GN combination could resist H_2_O_2_-induced apoptosis *in vitro*.

### GN Combination Activated the AMPK/mTOR/Nrf2 Pathways in H_2_O_2_-Induced Human Umbilical Vein Endothelial Cells

**FIGURE 8:**
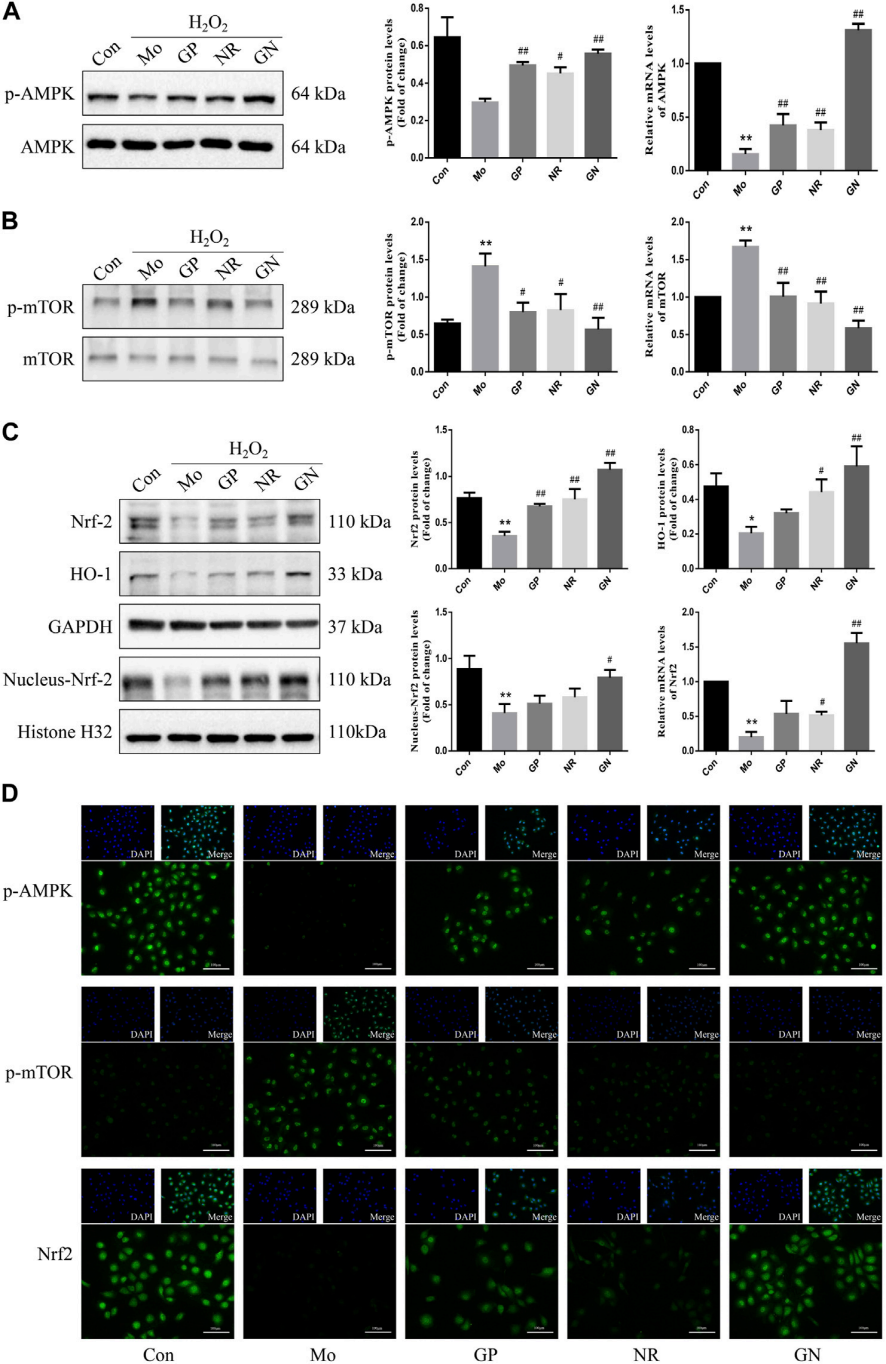
GN combination activated AMPK/mTOR/Nrf2 pathway in H_2_O_2_-induced HUVECs. The relative protein and mRNA levels of AMPK. **(A)**, mTOR **(B)**, and Nrf2. **(C)** were determined by WB and RT-PCR. The representative fluorescent staining images of p-AMPK, *p*-mTOR and Nrf2. **(D)** (scale bar, 100 μm, 200×). All the data are presented as the mean ± SD, *n* = 3. *p < 0.05 vs. control group, **p < 0.01 vs. control group, ^#^ *p* < 0.05 vs. model group, ^##^ *p* < 0.01 vs. model group.

Next, we explored the effects of GN combination on AMPK/mTOR/Nrf2 pathways in H_2_O_2_-induced HUVECs. Our study found that the expression of mRNA and phosphorylated protein of AMPK decreased, while that of mTOR increased in the model group compared to the normal control group; GP, NR, and GN combination could reverse these findings, with the effect of GN combination being most obvious (Figures 8A,B). At the same time, we also found that the total protein and mRNA expressions of Nrf2 and HO-1 were decreased in HUVECs induced by H_2_O_2_ alone, but this decrease was inhibited by GN combination. Moreover, the expression of nuclear Nrf2 was also increased under the intervention of GN combination (Figure 8C). Immunofluorescence staining also confirmed the above WB results (Figure 8D). Therefore, GN combination could activate the AMPK/mTOR and Nrf2/HO-1 signaling pathways in H_2_O_2_-induced HUVECs.

### GN Combination Inhibited Inflammation and Apoptosis in Human Umbilical Vein Endothelial Cells by Activating AMPK

**FIGURE 9:**
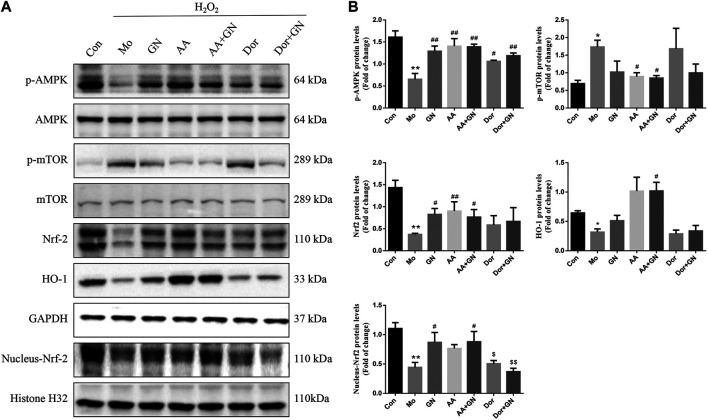
GN combination inhibited inflammation and apoptosis in HUVECs by activating AMPK. The cells were randomly divided into seven groups: Normal control group (Con), Model group (Mo), Geniposide + Notoginsenoside R1 treatment group (GN), AICAR treatment group (AA), Geniposide + Notoginsenoside R1 + AICAR treatment group (AA + GN), Dorsomorphin treatment group (Dor) and Geniposide + Notoginsenoside R1 + Dorsomorphin treatment group (Dor + GN). The relative protein levels of AMPK, mTOR, Nrf2 and HO-1. **(A)** were determined by WB, and the relative protein levels were normalized to GAPDH. **(B)**. All the data are presented as the mean ± SD, n = 3. *p < 0.05 vs. control group, **p < 0.01 vs. control group, ^#^ *p* < 0.05 vs. model group, ^##^ *p* < 0.01 vs. model group.

Based on the above results, we further explored whether the inhibition of inflammatory response and apoptosis by GN binding is related to AMPK/mTOR/Nrf2 signal pathway. Therefore, HUVECs were pretreated with AICAR (AMPK agonist) or dorsomorphin (AMPK inhibitor). The results showed that AICAR could promote the phosphorylation of AMPK and Nrf2 proteins and inhibit the phosphorylation of mTOR protein, while dorsomorphin could inhibit the phosphorylation of AMPK and Nrf2 proteins and promote the phosphorylation of mTOR protein (Figure 9). Similarly, dorsomorphin could also inhibit the expression of nuclear Nrf2 protein. These results suggest that AMPK could likely be an upstream regulator of mTOR and Nrf2.

**FIGURE 10:**
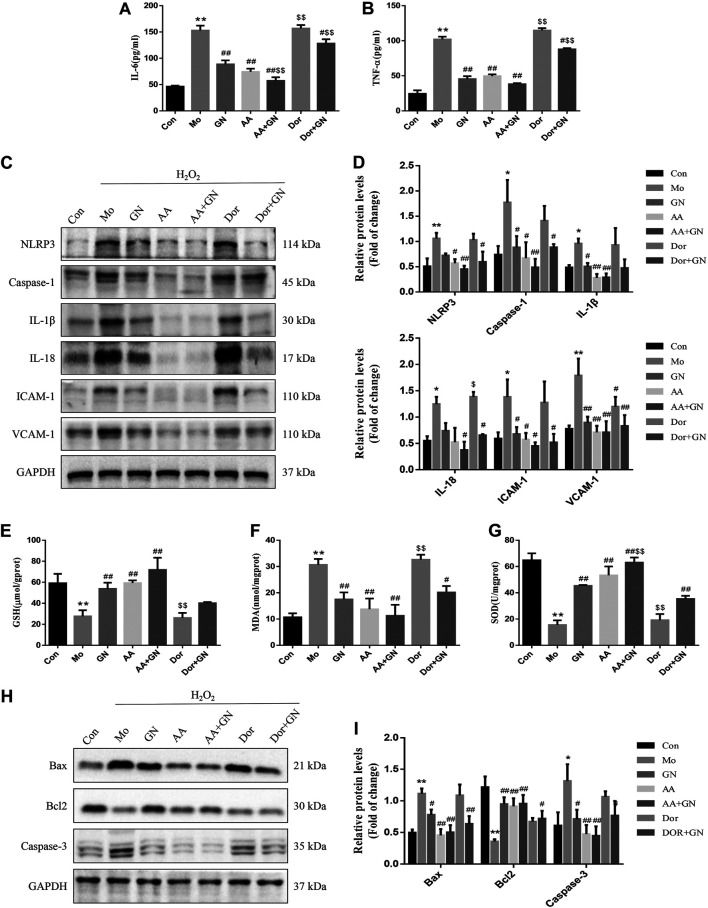
GN combination inhibited inflammation and apoptosis in HUVECs by activating AMPK. The concentration of IL-6. **(A)** and TNF-α. **(B)** in cell supernatants. The expression of NLRP3, caspase-1, IL-1β, IL-18, ICAM-1, VCAM-1, and GAPDH in HUVECs was determined by WB. **(C)**, and the relative protein levels were normalized to GAPDH **(D)**. The concentration of GSH. **(E)**, MDA. **(F)** and SOD **(G)** in cell supernatants. The expression of Bax, Bcl2, caspase-3 and GAPDH in HUVECs was determined by WB. **(H)**, and the relative protein levels were normalized to GAPDH. **(I)**. All the data are presented as the mean ± SD, *n* = 3. *p < 0.05 vs. control group, **p < 0.01 vs. control group, ^#^ *p* < 0.05 vs. model group, ^##^ *p* < 0.01 vs. model group.

We also investigated whether GN combination could play a role in the H_2_O_2_-induced inflammatory response in HUVECs by regulating the AMPK pathway. The results of WB showed that compared with the model group, the GN, AICAR, and AICAR + GN groups showed reduced expression of NLRP3, caspase-1, IL-1β, IL-18, ICAM-1, and VCAM-1 in H_2_O_2_-induced HUVECs; this effect was especially seen in the AICAR + GN group. On the other hand, dorsomorphin increased the expression of the above proteins in H_2_O_2_-induced HUVECs, regardless of the presence or absence of GN combination (Figures 10C,D). AICAR pretreatment could significantly reduce the concentrations of IL-6 and TNF-α in the supernatant of H_2_O_2_-induced HUVECs, which was the same as that observed in the GN group (Figures 10A,B). In addition, the activities of GSH and SOD in HUVECs treated with H_2_O_2_ for 24 h were significantly decreased and the concentration of MDA was increased; this effect was not seen in HUVECs treated with GN or AICAR (Figures 10E–G). Importantly, treatment with AICAR or AICAR + GN combination significantly inhibited the expression of Bax and caspase-3 protein and increased the expression of Bcl2, especially in the AICAR + GN group. However, the expression of these proteins in dorsomorphin-treated HUVECs was the same as that in the model group, although treatment with GN combination alleviated this effect (Figures 10H,I). These results suggest that GN combination plays a key role in H_2_O_2_-induced inflammatory response and apoptosis of HUVECs by activating the AMPK/mTOR/Nrf2 signaling pathway.

## Discussion

Atherosclerosis is a chronic vascular disease caused by multiple factors and involves pathological processes such as inflammation, oxidative stress, apoptosis, lipid accumulation, autophagy and cellular proliferation and migration. However, the most characteristic pathological aspect of AS is the persistent and long-term inflammatory response and apoptosis of the blood vessel intima, which can eventually lead to the formation of a necrotic core and plaque instability (Wolf and Ley, 2019). Moreover, endothelial cells are the main components of the vascular intima and the first vital barrier to protect blood vessels (Onat et al., 2011). Therefore, inhibiting the inflammatory response and apoptosis of vascular endothelial cells is essential to alleviate the occurrence and development of AS. GP and NR1 are active compounds extracted from *G. jasminoides Ellis* and notoginseng, respectively. It has been proven that GP or NR used alone can alleviate the occurrence and development of AS (Jia et al., 2014; Xu et al., 2020). GN combination is a patented and approved TCM drug for the prevention and treatment of AS, whose effect is better than that of GP or NR used alone. However, the specific mechanism of action for the treatment of AS is not yet clear. It is well-known that ApoE^**−/−**^ mice is used to reduplicate the atherosclerosis model, and the vascular endothelial cell injury model is mostly HUVECs induced by ox-LDL or H_2_O_2_ (Li et al., 2004; Getz and Reardon, 2016; Pankratz et al., 2018; Huang et al., 2019). In this study, we used the above methods to establish the model respectively. The result showed that, the body weight, blood glucose and serum levels of TC, TG, LDL-C, and HDL-C of the HFD-fed ApoE^**−/−**^ mice were significantly higher than those of the control mice. Further, obvious plaques were observed in the aortic sinus of the former group after histopathological staining. On the other hand, the GN combination could reduce the body weight, blood glucose levels, and serum levels of TC, TG, and LDL-C levels of mice, increase HDL-C levels, and reduce the formation of AS plaques; the effects of GN combination were better than those of GP or NR alone. Thus, GN combination can improve the HFD-induced pathological changes in AS.

The inflammatory response of vascular endothelial cells is involved in every stage of the occurrence and development of AS (Ding et al., 2020). When arterial endothelial cells are activated by hyperlipidemia, oxidative stress, and other factors, they can secrete several inflammatory factors and adhesion molecules, leading to monocyte adhesion and endothelial penetration, thereby promoting the occurrence and development of AS. Therefore, inflammatory factors and adhesion molecules are considered important predictors for the occurrence and development of AS. In this study, we found that in HFD-fed ApoE^**−/−**^ mice and H_2_O_2_-induced HUVECs, the secretion levels of IL-6, TNF-α, IL-8 and IL-15 and the expression levels of ICAM-1 and VCAM-1 increased, indicating that both HFD and H_2_O_2_ can induce inflammation *in vivo* and *in vitro,* respectively. In addition, GN combination could reduce the secretion of IL-6 and TNF-α and inhibit the expression of ICAM-1 and VCAM-1 *in vitro* and *in vivo*. Moreover, the results of our *in vitro* experiments showed that the number of THP-1 cells adhered to endothelial cells increased significantly after HUVECs were induced by H_2_O_2_ for 24 h; however, GN combination significantly reduced the number of adherent THP-1 cells, which further confirmed that GN combination could alleviate the H_2_O_2_-induced inflammatory response.

The activated NLRP3 inflammasome enhances the inflammatory response by promoting the production and secretion of caspase-1, IL-1β, IL-18, and other key downstream cytokines, in order to maintain the body’s innate and specific immunity (Mangan et al., 2018). Studies have confirmed that the activation of NLRP3 inflammasome plays a key role in the occurrence and development of AS (Mangan et al., 2018). When blood lipids, oxidative stress, and inflammatory mediators increase, the NLRP3 inflammasome pathway is activated, and the levels of apoptosis-related proteins, NLRP3, caspase-1, IL-1β, and IL-18 are up-regulated, thus increasing the expression of inflammatory and adhesion factors to promote plaque formation (Janoudi et al., 2016; Liu et al., 2018). In this study, we found that compared with the control group, the model group showed increased expression levels of NLRP3, caspase-1, IL-1β, and IL-18 in the aortic tissue; however, the expression of NLRP3 inflammasome-related components was significantly inhibited by GN combination. In H_2_O_2_-induced HUVECs, the expression of related proteins and the regulation of GN combination were consistent with the *in vivo* results of HFD-fed mice. Therefore, GN combination can inhibit AS inflammatory response by inhibiting the expression of NLRP3 inflammasome-related proteins.

Nowadays, Vascular endothelial cell apoptosis is considered an independent risk factor of atherosclerosis (Perez et al., 2015). In particular, Caspase-3, Bax, and Bcl2 are key proteins related to cellular apoptosis, which can be activated through internal and external apoptotic pathways, and their expression directly reflects the degree of cellular apoptosis (Pal et al., 2016; Suresh et al., 2019). Several studies have shown that hyperlipidemia; high levels of homocysteine (Hcy), Lipopolysaccharide (LPS), glucose; oxidative stress; and other cardiovascular risk factors can accelerate endothelial cell apoptosis and cause cardiovascular diseases (Zhang et al., 2017; Hou et al., 2019; Yi and Gao, 2019; Wu et al., 2020). In this study, HFD led to increased serum MDA concentration in ApoE^**−/−**^ mice and decreased SOD and GSH activity. The results in the supernatant of H_2_O_2_-induced HUVECs are consistent with the *in vivo* results; thus, H_2_O_2_ could significantly induce apoptosis and increase the level of ROS in HUVECs. The GN combination could significantly reduce the apoptosis rate of HUVECs and the concentration of MDA and ROS and increase the activity of SOD and GSH better than GP or NR alone. Furthermore, our study found that compared with the control group, the model group showed increased expression of Bax, caspase-3, NOX2 and p22phox and decreased expression of Bcl2, both in the aortic tissue of HFD-fed ApoE^**−/−**^ mice and in H_2_O_2_-induced HUVECs. Treatment with GN combination could significantly reduce the expression of Bax, caspase-3, NOX2 and p22phox protein and increase the expression of Bcl2 protein. In word, the GN combination could inhibit the apoptosis of vascular endothelial cells by regulating the Bax/Bcl2/caspase-3 axis.

Finally, to study the specific molecular mechanism by which GN combination inhibits inflammation and apoptosis of AS, we examined the role of the AMPK/mTOR/Nrf2 signaling pathway. More and more evidence shows that AMPK plays an important role in regulating as plaque stability. Activated AMPK can reduce the level of IL-6 and IL-1 β and decreased the activation of NLRP3 inflammasome, while dorsomorphin antagonized the effect of AMPK (Zhang et al., 2020). In addition, AMPK can improve proliferation and apoptosis in HUVECs induced by ox-LDL, reduce the production of ROS and up regulate the expression of antioxidant enzymes (Sun et al., 2017). Therefore, AMPK plays an important role in improving inflammation and apoptosis of AS. It has been reported that GP was capable to protect mice and cells from nonalcoholic fatty liver disease-induced oxidative stress and inflammation, which were mostly depend on up-regulating the protein expression of Nrf2/HO-1 and AMPK signalling pathways (Shen et al., 2020). NR pretreatment attenuated myocardium injury and cardiac malfunction after myocardial ischemia reperfusion via inhibiting ROCK and enhancing the expression of AMPK (He et al., 2014). This results indicate that AMPK is an important target for GP to antagonize inflammation and oxidative stress. At the same time, our results confirm that compared with the model group, GN combination could significantly promote the phosphorylation of AMPK protein, inhibit the phosphorylation of mTOR protein, and induce the expression of Nrf2 and HO-1 proteins, both *in vivo* and *in vitro*. More importantly, pretreatment of HUVECs with AICAR could significantly inhibit the phosphorylation of mTOR protein, induce the expression of Nrf2 and HO-1, inhibit the expression of NLRP3 inflammasome-related and apoptosis-related proteins, and inhibit inflammation and apoptosis. The above effects could be reversed by administering dorsomorphin. These results show that AMPK is upstream of the mTOR and Nrf2 signals, and the AMPK/mTOR/Nrf2 signaling pathway is involved in the mechanism by which GN combination inhibits inflammation and apoptosis of vascular endothelial cells.

## Conclusion

In short, our study confirmed that GN combination is superior to both GP and NR used alone in alleviating the occurrence and development of AS. GN combination can improve body weight and blood glucose and blood lipid levels, inhibit inflammatory response and apoptosis, and reduce plaque formation, which may be related to the activation of the AMPK/mTOR/Nrf2 signaling pathway. We believe that our results can strengthen the evidence for future use of GN combination prevention and treatment of AS clinically.

## Data Availability

The raw data supporting the conclusion of this article will be made available by the authors, without undue reservation.
