# Supplementary material for: Geniposide Combined With Notoginsenoside R1 Attenuates Inflammation and Apoptosis in Atherosclerosis via the AMPK/mTOR/Nrf2 Signaling Pathway
Source: Front Pharmacol. 2021 Jul 7;12:687394. doi: 10.3389/fphar.2021.687394 (PMC8293676; doi:10.3389/fphar.2021.687394)
Supplement: Supplementary file 1 [file DataSheet1.docx]

**Geniposide combined with notoginsenoside R1 attenuates inflammation and apoptosis in atherosclerosis via the AMPK/mTOR/Nrf2 signaling pathway**

**Xiaoyu Liu^1†^, Yuling Xu^2^, Saibo Cheng^1^, Xinghong Zhou^1^, Fenghua Zhou^1^, Peikun He^1^, Fang Hu^1^, Lifang Zhang^1^, Yuyao Chen^1^, Yuyua Jia^1*^**

^1^College of traditional Chinese medicine, Southern Medical University, Guangzhou, China

^2^College of Health, Fujian Medical University, Fuzhou, China

*** Correspondence:**Yuhua Jia
[yuhuajia_smu@126.com](mailto:yuhuajia_smu@126.com)


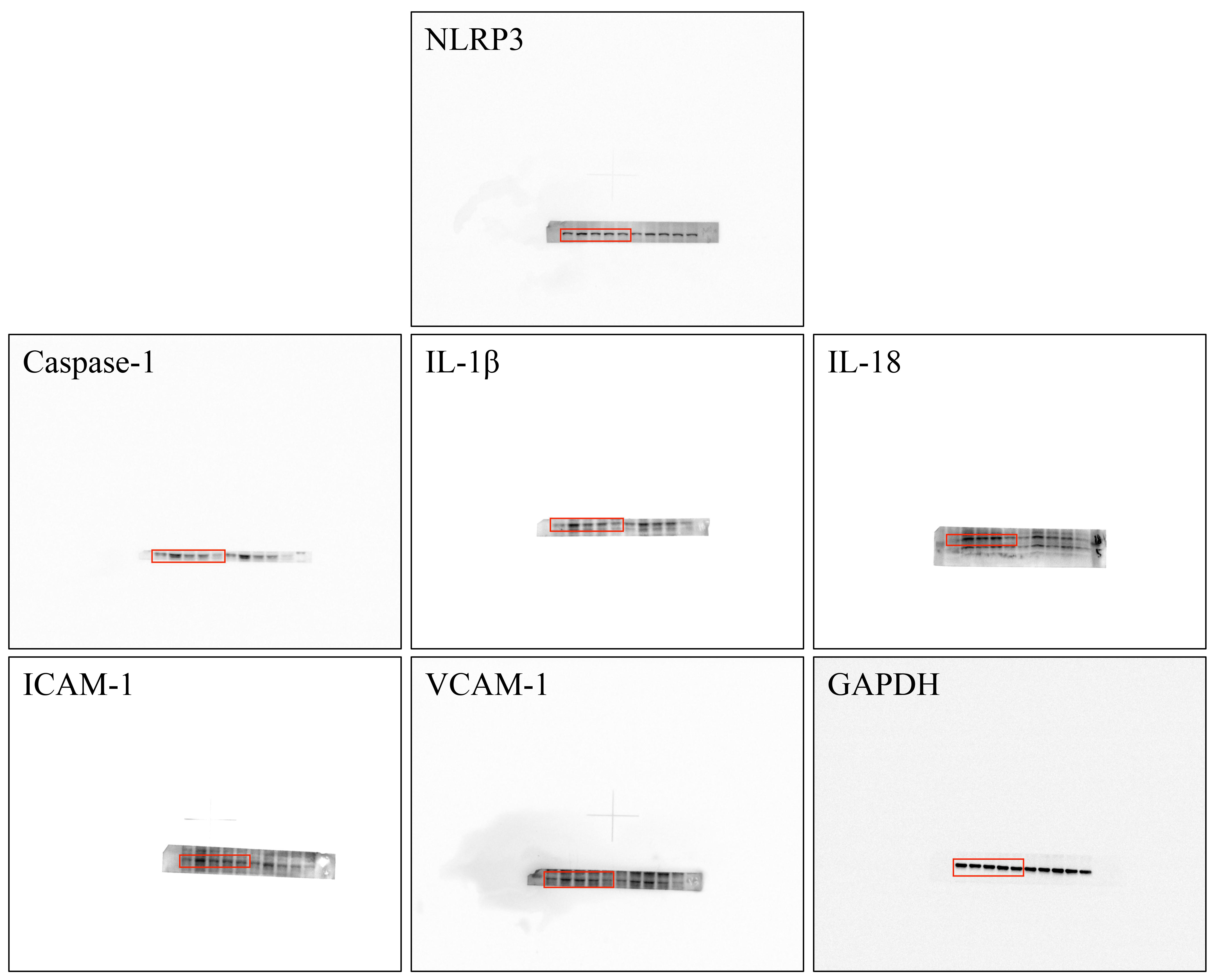


**Figure S1.** The uncropped western blot results of NLRP3, Caspase-1, IL-1β, IL-18, ICAM-1, VCAM-1 and GAPDH in aortic tissues of ApoE^-/-^ mice from different treatment groups.


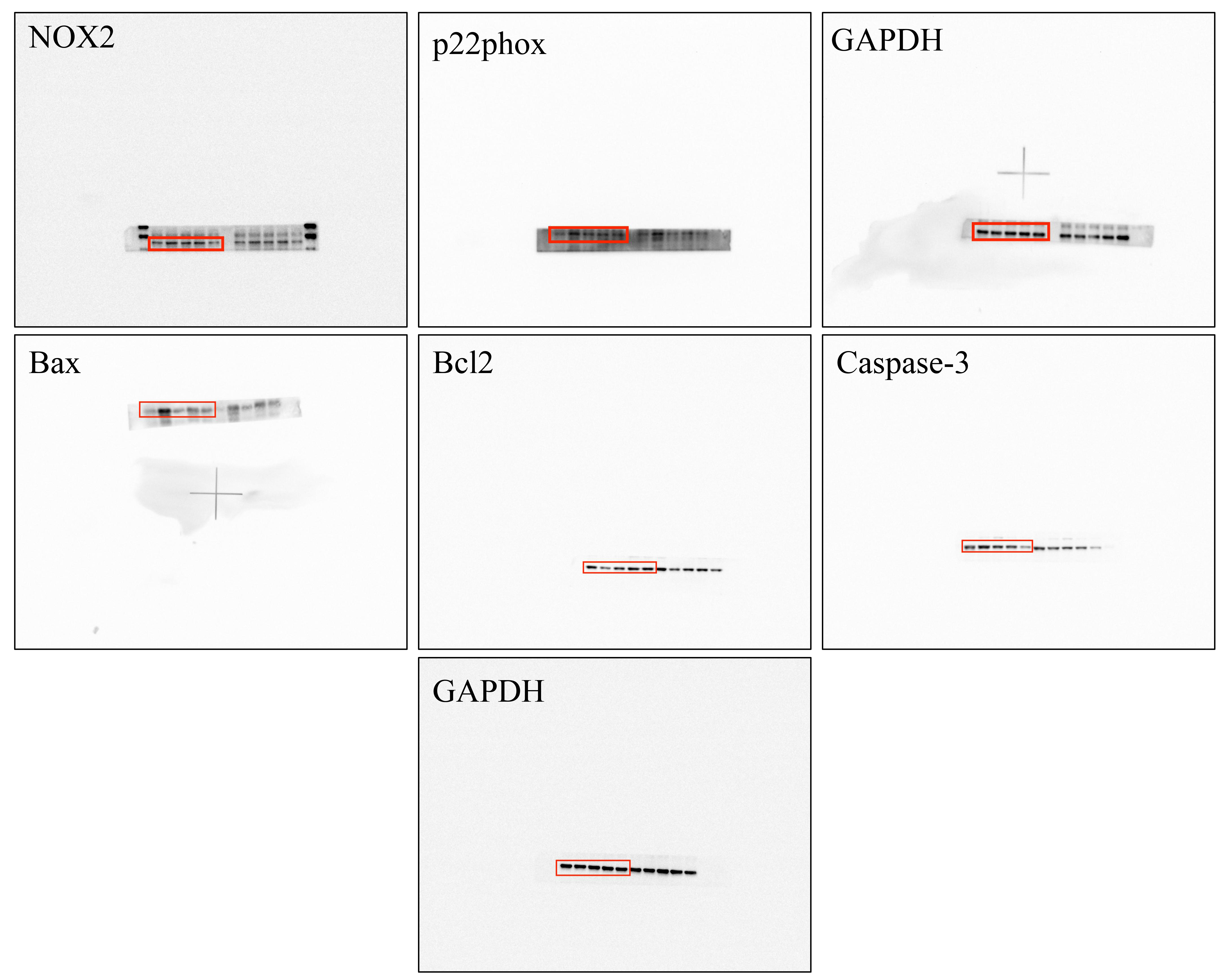


**Figure S2.** The uncropped western blot results of NOX2, p22phox, Bax, Bcl2, Caspase-3 and GAPDH in aortic tissues of ApoE^-/-^ mice from different treatment groups.


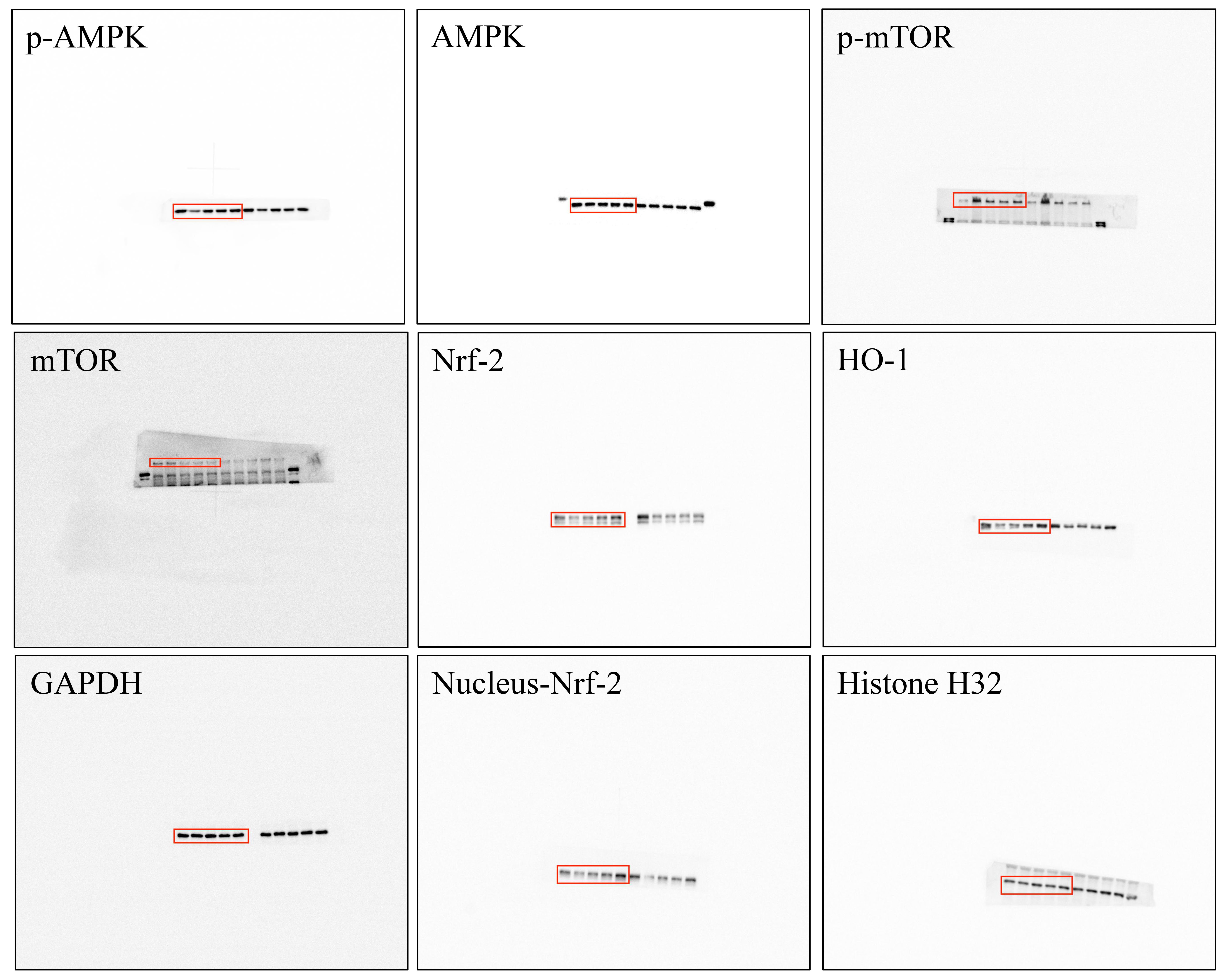


**Figure S3.** The uncropped western blot results of p-AMPK, AMPK, p-mTOR, mTOR, Nrf-2, HO-1, GAPDH, Nucleus-Nrf-2 and Histone H32 in aortic tissues of ApoE^-/-^ mice from different treatment groups.

.


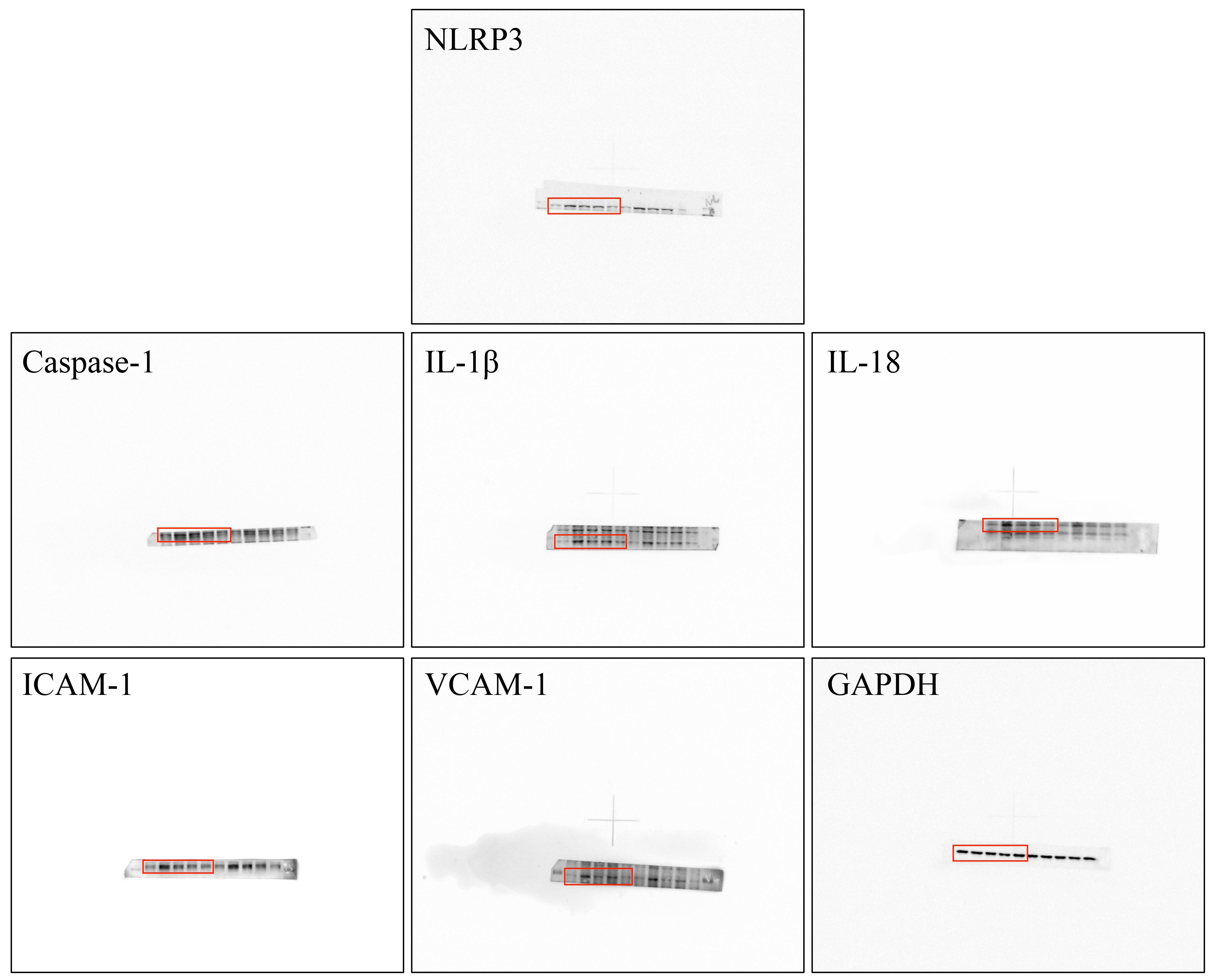


**Figure S4.** The uncropped western blot results of NLRP3, Caspase-1, IL-1β, IL-18, ICAM-1, VCAM-1 and GAPDH in HUVECs treated with GN combination from different treatment groups.


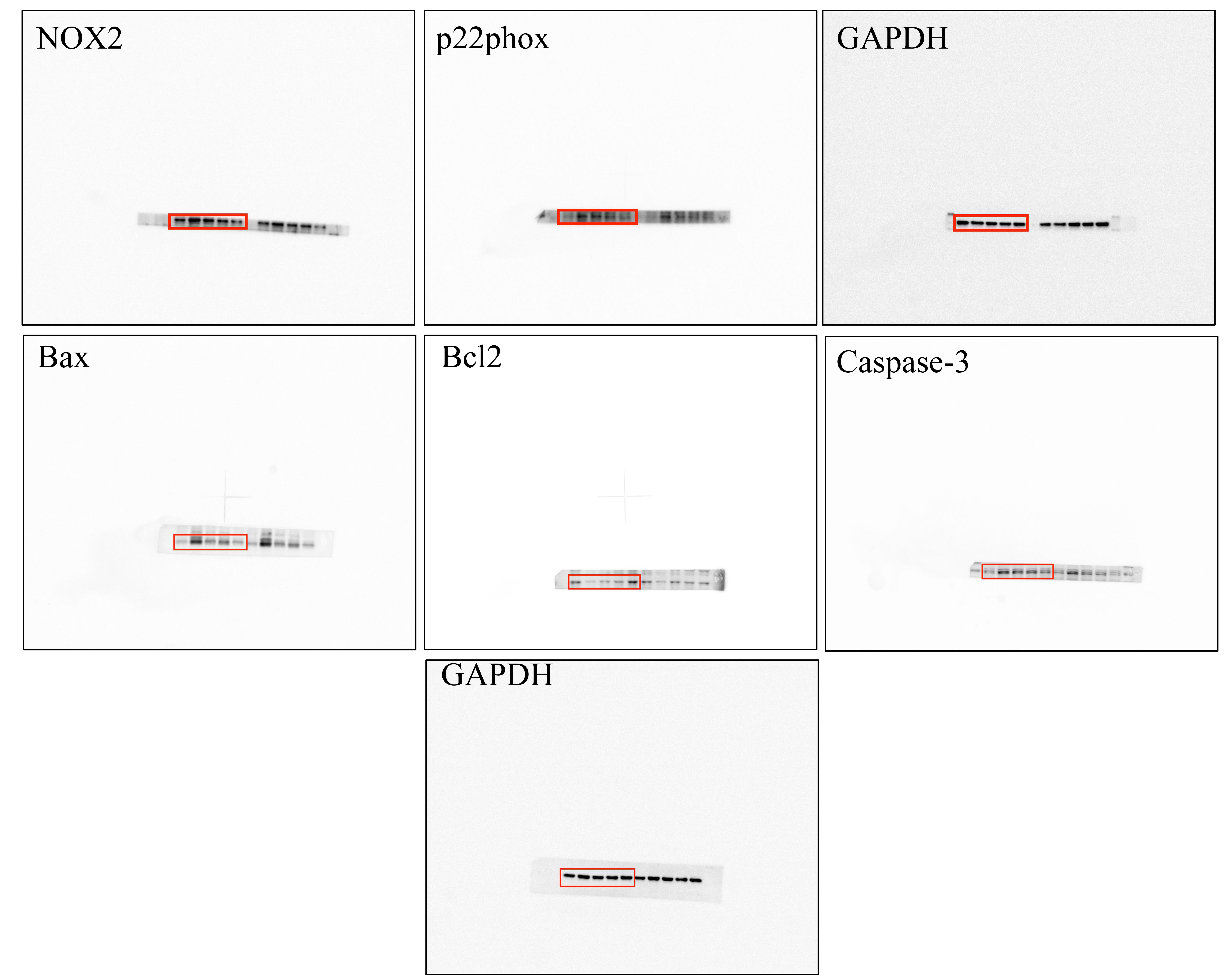


**Figure S5.** The uncropped western blot results of NOX2, p22phox, Bax, Bcl2, Caspase-3 and GAPDH in HUVECs treated with GN combination from different treatment groups.


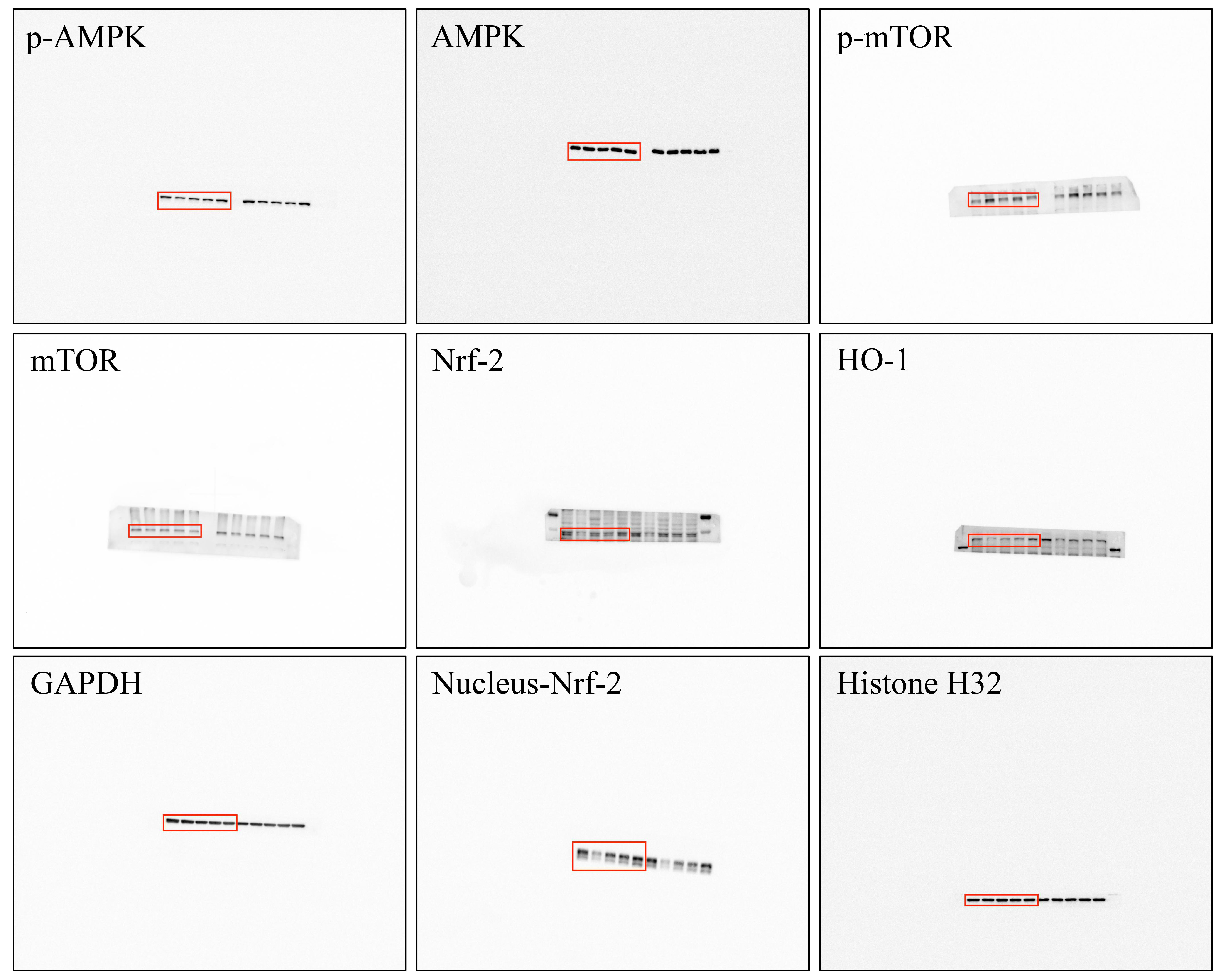


**Figure S6.** The uncropped western blot results of p-AMPK, AMPK, p-mTOR, mTOR, Nrf-2, HO-1, GAPDH, Nucleus-Nrf-2 and Histone H32 in HUVECs treated with GN combination from different treatment groups.


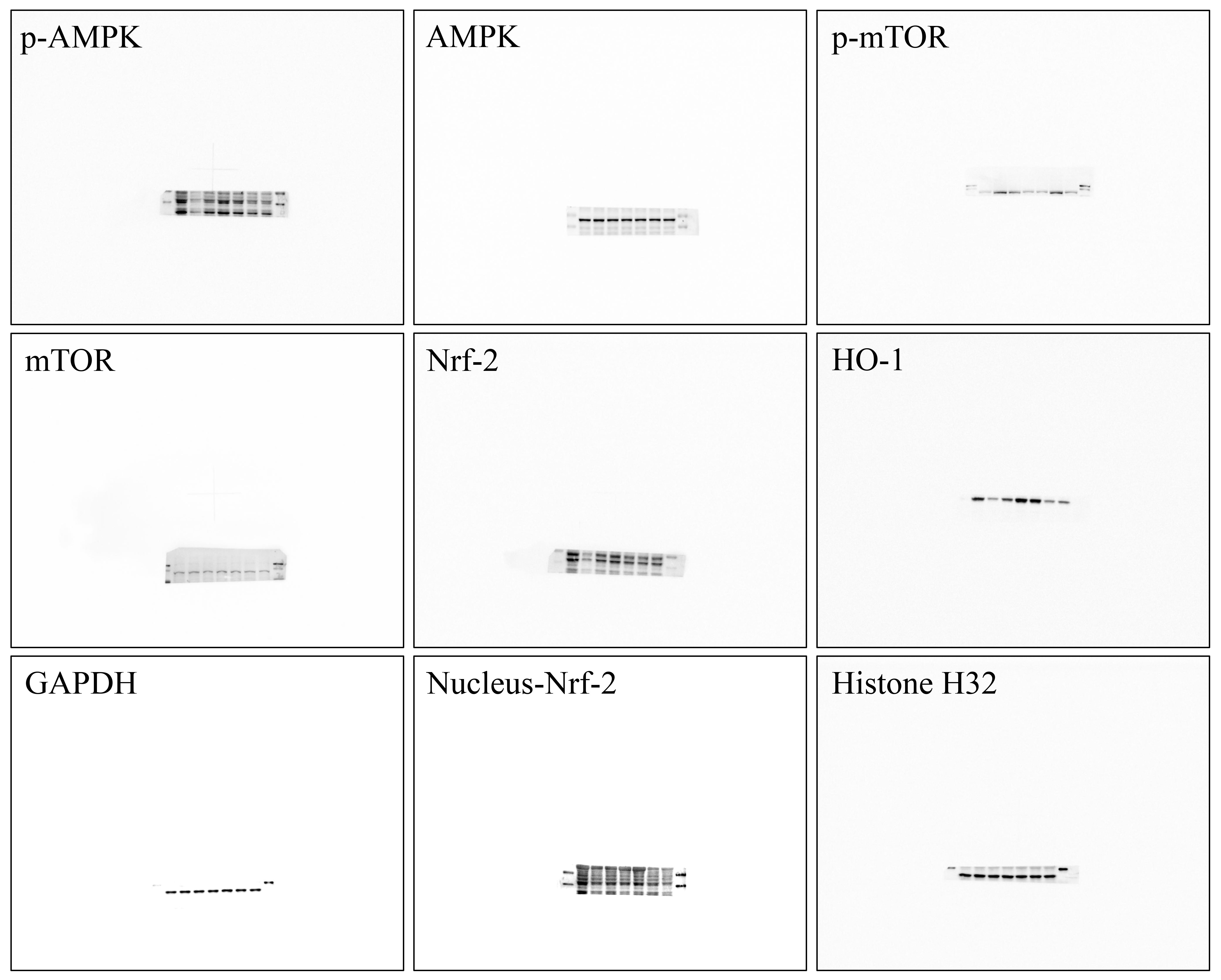


**Figure S7.** The uncropped western blot results of p-AMPK, AMPK, p-mTOR, mTOR, Nrf-2, HO-1, GAPDH, Nucleus-Nrf-2 and Histone H32 in HUVECs treated with AICAR or dorsomorphin from different treatment groups.


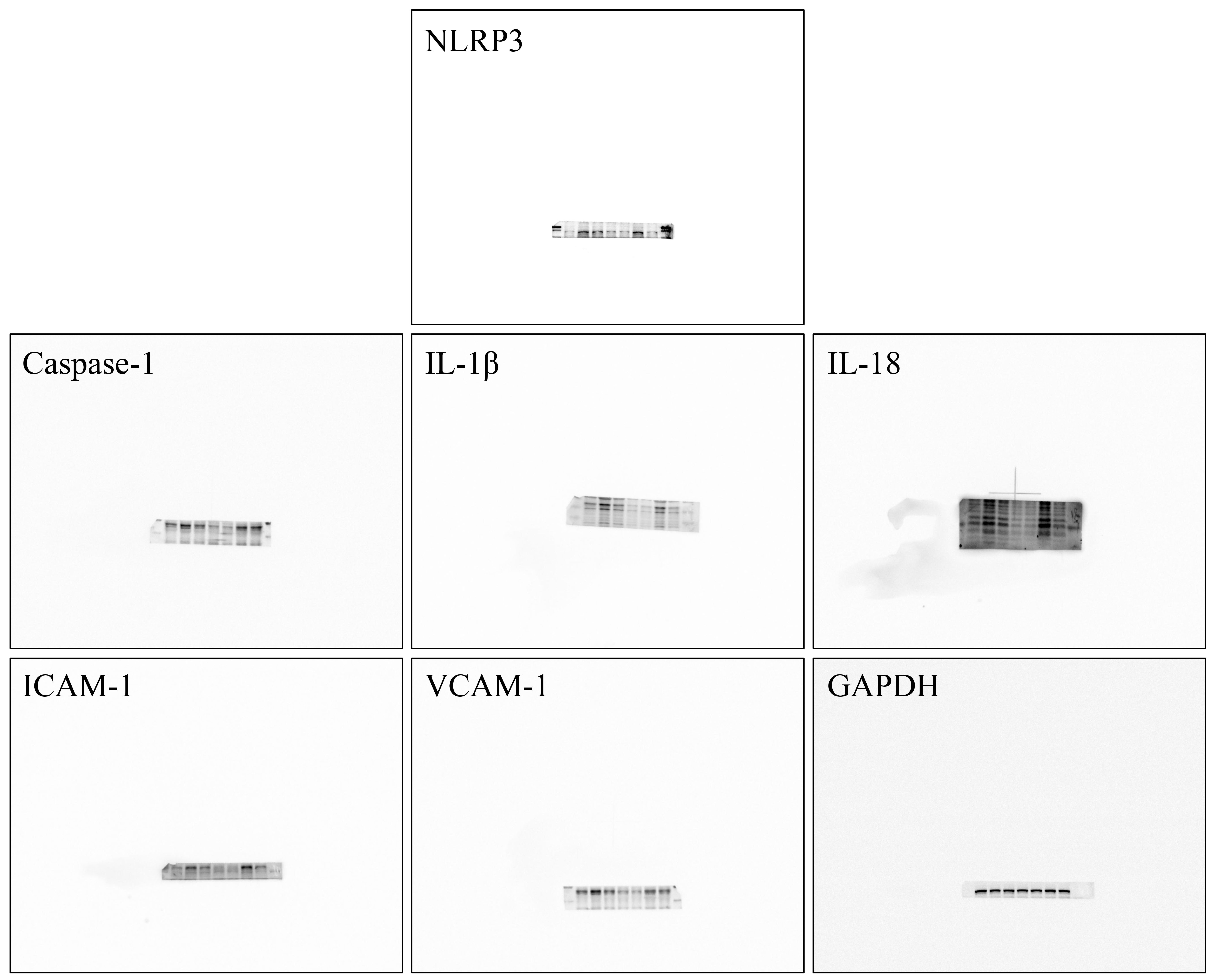


**Figure S8.** The uncropped western blot results of NLRP3, Caspase-1, IL-1β, IL-18, ICAM-1, VCAM-1 and GAPDH in HUVECs treated with AICAR or dorsomorphin from different treatment groups.


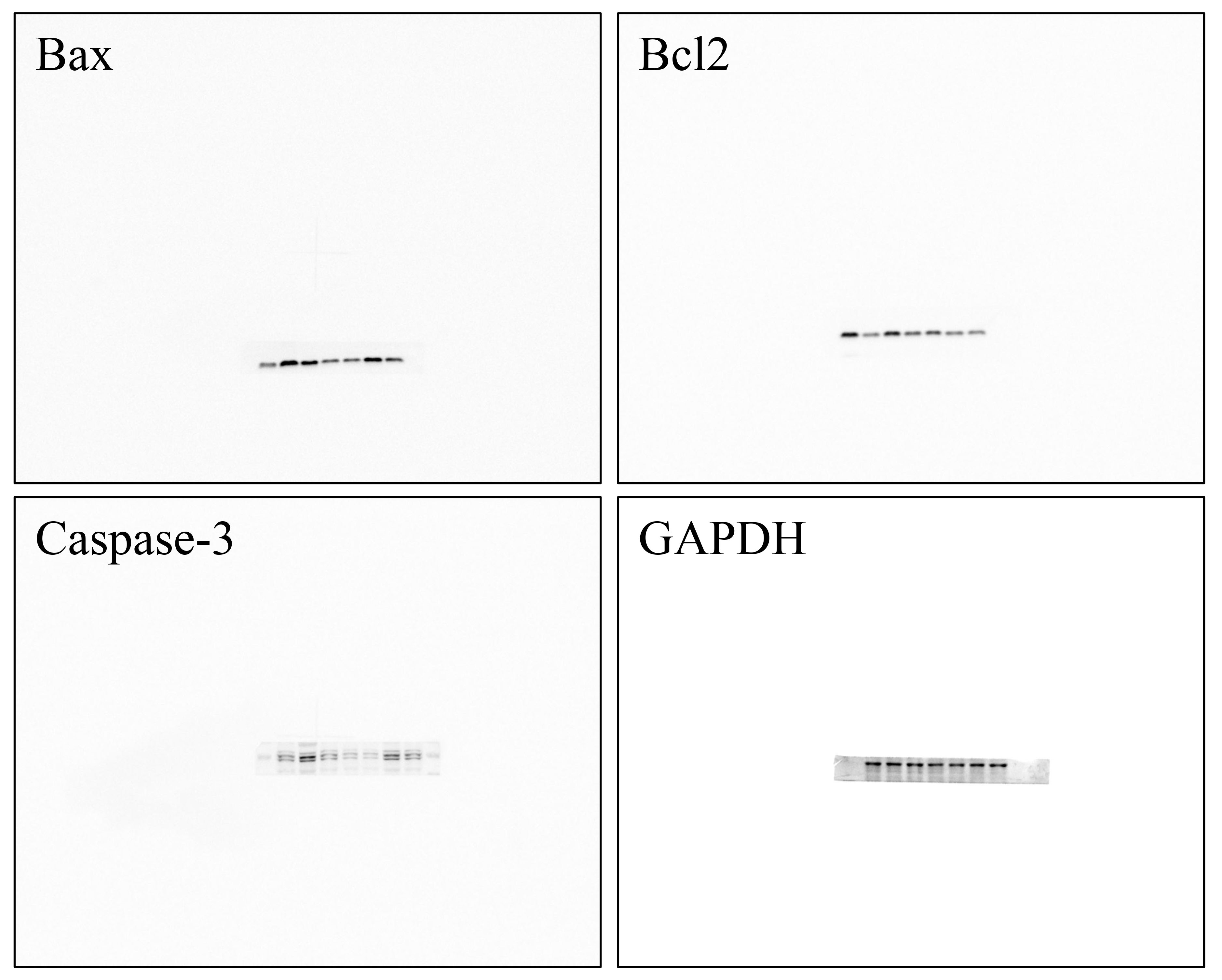


**Figure S9.** The uncropped western blot results of Bax, Bcl2, Caspase-3 and GAPDH in HUVECs treated with AICAR or dorsomorphin from different treatment groups.
